# Life History and Smoltification Shape Genomic Signatures of Thermal Tolerance in Chinook Salmon

**DOI:** 10.1111/mec.70233

**Published:** 2026-01-12

**Authors:** David C. H. Metzger, Timothy M. Healy, Kyle Wellband, Patricia M. Schulte

**Affiliations:** ^1^ Department of Zoology The University of British Columbia Vancouver British Columbia Canada; ^2^ Pacific Biological Station, Fisheries and Oceans Canada Nanaimo British Columbia Canada; ^3^ Pacific Science Enterprise Centre, Fisheries and Oceans Canada West Vancouver British Columbia Canada

## Abstract

Temperature is a key determinant of survival and distribution in ectothermic species, but how variation in thermal resilience is influenced by developmental transitions, life‐history strategies, and their interaction with population‐specific genomic variation is poorly understood. Using Chinook salmon ( *Oncorhynchus tshawytscha* ), an ecologically and culturally important species of conservation concern, we investigated how population‐specific life histories influence thermal tolerance and its underlying genomic architecture. We assessed thermal tolerance using critical thermal maximum (CT_max_), a nonlethal measure of acute thermal tolerance that determines the temperature at which fish can no longer maintain equilibrium during a thermal ramp. CT_max_ was measured in four populations representing two life‐history types, stream‐type and ocean‐type, that differ in freshwater residency and age at smoltification. Stream‐type populations exhibited lower CT_max_ than ocean‐type populations in both freshwater and saltwater, revealing consistent life‐history differences in thermal tolerance. Smoltification significantly reduced CT_max_ across all populations, indicating that physiological transformation for seawater readiness comes at a cost to thermal performance. Thermal tolerance was more variable in saltwater, highlighting the influence of environmental context on phenotypic expression. Although populations exhibited distinct genetic variants and expression profiles, all populations showed enrichment of common functional pathways, including the unfolded protein response and ion transport. These findings suggest that similar physiological outcomes are achieved through distinct regulatory architectures across life‐history types and developmental stages. Together, our results provide insight into the polygenic nature of thermal tolerance in Chinook salmon, emphasising how life history, environment and genetic background interact to shape resilience to thermal stress.

## 1 Introduction

Pacific salmon populations are facing increasing threats from rapid environmental change. Across the Pacific, climate shifts have contributed to widespread declines in salmon survival and productivity, with negative effects observed across both freshwater and marine life stages (Crozier et al. 2021). Projected reductions in snowpack, groundwater availability and altered streamflow regimes are expected to further disrupt thermal conditions in freshwater ecosystems, which are essential for spawning, early development and migration (Brown 2002; Cunningham et al. 2018; Wieder et al. 2022). These environmental stressors are being intensified by anthropogenic activity, which poses an overarching threat to the viability of salmon populations across their range (Abdul‐Aziz et al. 2011; M. Healey 2011; Peterman and Dorner 2012). In southern British Columbia (BC), these challenges are of particular concern for Chinook salmon ( *Oncorhynchus tshawytscha* ), a species of significant ecological, economic and cultural value. Hatchery programs, such as those operated by the Canadian Department of Fisheries and Oceans (DFO) Salmonid Enhancement Program (SEP), have supported Chinook salmon populations across the region since the 1960s. Across BC, these hatcheries maintain multiple populations that differ in genetic background and life‐history strategy, providing a valuable framework to investigate how populations may differ in their physiological sensitivity to climate‐related stressors. In particular, early‐life stages are especially vulnerable to elevated temperatures in freshwater habitats, making thermal performance a key trait for understanding population‐specific responses to environmental change (Crozier et al. 2021).

Chinook salmon exhibit two distinct juvenile life‐history strategies, stream‐type and ocean‐type, that differ in the timing of ocean entry and the duration of freshwater residence. Ocean‐type juveniles typically migrate to the ocean within their first year, often utilising estuarine habitats before entering the ocean (M. C. Healey 1991; Waples et al. 2004). In contrast, stream‐type juveniles remain in freshwater for a year or more prior to saltwater entry (M. C. Healey 1991). The migratory strategies associated with these lineages likely influence the thermal experiences of juveniles, with stream‐type juveniles remaining in freshwater for extended periods, where extreme heat events in the freshwater environment can significantly impact their growth and survival during early life stages. Therefore, understanding how these differing freshwater residency strategies are reflected in the underlying genomic and molecular processes may provide key new insights into the physiological resilience and adaptive potential of Chinook salmon (Crozier et al. 2021).

A key developmental process that varies across life‐history types is the timing of smoltification. During smoltification, juveniles undergo extensive physiological changes to transition from freshwater to marine environments. These changes include restructuring of osmoregulatory systems, endocrine shifts and adjustments in metabolism, immunity and behaviour (Bernard et al. 2020; McCormick et al. 1998). Ocean‐type Chinook salmon typically initiate smoltification 60–90 days postemergence, while stream‐type fish delay this transition until their second spring, or even later. Temperature strongly influences the timing and pace of smoltification, with warmer conditions accelerating the onset of this process (Handeland et al. 2004; Zydlewski et al. 2005). However, high temperatures can also have deleterious effects, such as limiting the physiological window of saltwater tolerance, reducing swimming speed, and even inducing ‘desmoltification’, where smolts lose their saltwater adaptation (Bernard et al. 2020; Handeland et al. 2004). While much is known about the temperature sensitivity of smoltification, less is understood about how these temperature‐dependent shifts affect the resilience of different life histories, particularly in response to extreme heat events. Given that climate change is altering stream temperatures and flow regimes, leading to both chronic and acute thermal challenges, understanding how smoltification influences the thermal tolerance and physiological resilience of Chinook salmon is critical for assessing the adaptability of ocean‐type and stream‐type populations (Mayer et al. 2024).

Thermal sensitivity differs across Chinook salmon life stages and populations, shaped in part by variation in life history and rearing environments (Oyinlola et al. 2024; Van Wert et al. 2023; Zillig, Fitzgerald, et al. 2023; Zillig, Lusardi, et al. 2023). Temperature affects key physiological processes including growth, metabolism and migratory performance, with evidence of population‐specific thermal thresholds (Mayer et al. 2024; Schulte and Healy 2022). Stream‐type populations, with prolonged freshwater residence, may be especially vulnerable to warming streams and hydrological shifts. Ocean‐type juveniles, while spending less time in freshwater, may still face risks if access to productive estuarine habitats is reduced. In this study, we examine how smoltification timing and life‐history strategy influence thermal tolerance across Chinook salmon populations, with the goal of understanding how developmental trajectories might mediate responses to climate‐driven environmental change.

Genomic approaches provide a powerful framework for investigating thermal tolerance in salmon by linking environmental exposures to underlying molecular processes. Life‐history variation, including differences in freshwater residency and the timing of smoltification, shapes the thermal environments juveniles experience, yet how these divergent trajectories have influenced underlying physiological and genomic architecture remains poorly understood. Comparative genomic approaches such as genome‐wide scans and transcriptomic profiling can identify loci and regulatory pathways associated with temperature stress responses (Gonen et al. 2024), helping to distinguish between heritable genetic variation, plasticity or their interaction (Benfey et al. 2024; Morgan et al. 2022). Population genomic analyses can also reveal adaptive divergence across watersheds and life‐history types, providing insight into the evolutionary basis of thermal resilience and informing conservation and hatchery management (Akbarzadeh et al. 2024; Bourret et al. 2013; Carvalheiro et al. 2025).

In this study, we integrate physiological and genomic approaches to investigate thermal tolerance in Chinook salmon populations from southern British Columbia. Our goal was to identify genetic and transcriptomic correlates of resilience to thermal stress, with a particular focus on differences between life‐history types and the effects of smoltification on thermal tolerance.

To address these goals, we ask four interconnected questions. First, do stream‐type and ocean‐type Chinook salmon differ in thermal tolerance, which we quantify using critical thermal maximum (CT_max_), a nonlethal measure of acute thermal tolerance determined by the temperature at which fish lose equilibrium during a thermal ramp? Second, can we identify genomic regions associated with increased thermal resilience, and are these associations consistent across populations with different life histories? Third, does baseline gene expression in gill tissue predict individual variation in CT_max_ among presmolt juveniles? And fourth, does the relationship between genotype and thermal tolerance shift across developmental stages and salinity environments? By addressing these questions, we aim to disentangle the developmental, physiological and genomic factors that shape thermal resilience in Chinook salmon, with implications for conservation under climate change.

## 2 Materials and Methods

### 2.1 Study Organisms

To investigate how different life histories influence the ability of Chinook salmon to tolerate high temperatures in freshwater and saltwater, we assessed acute thermal tolerance in two distinct life‐history types of Chinook salmon: The ocean‐type, which migrates to the sea within the first few months of life and the stream‐type, which spends one or more years in freshwater before migrating to the sea (M. C. Healey 1991).

**FIGURE 1:**
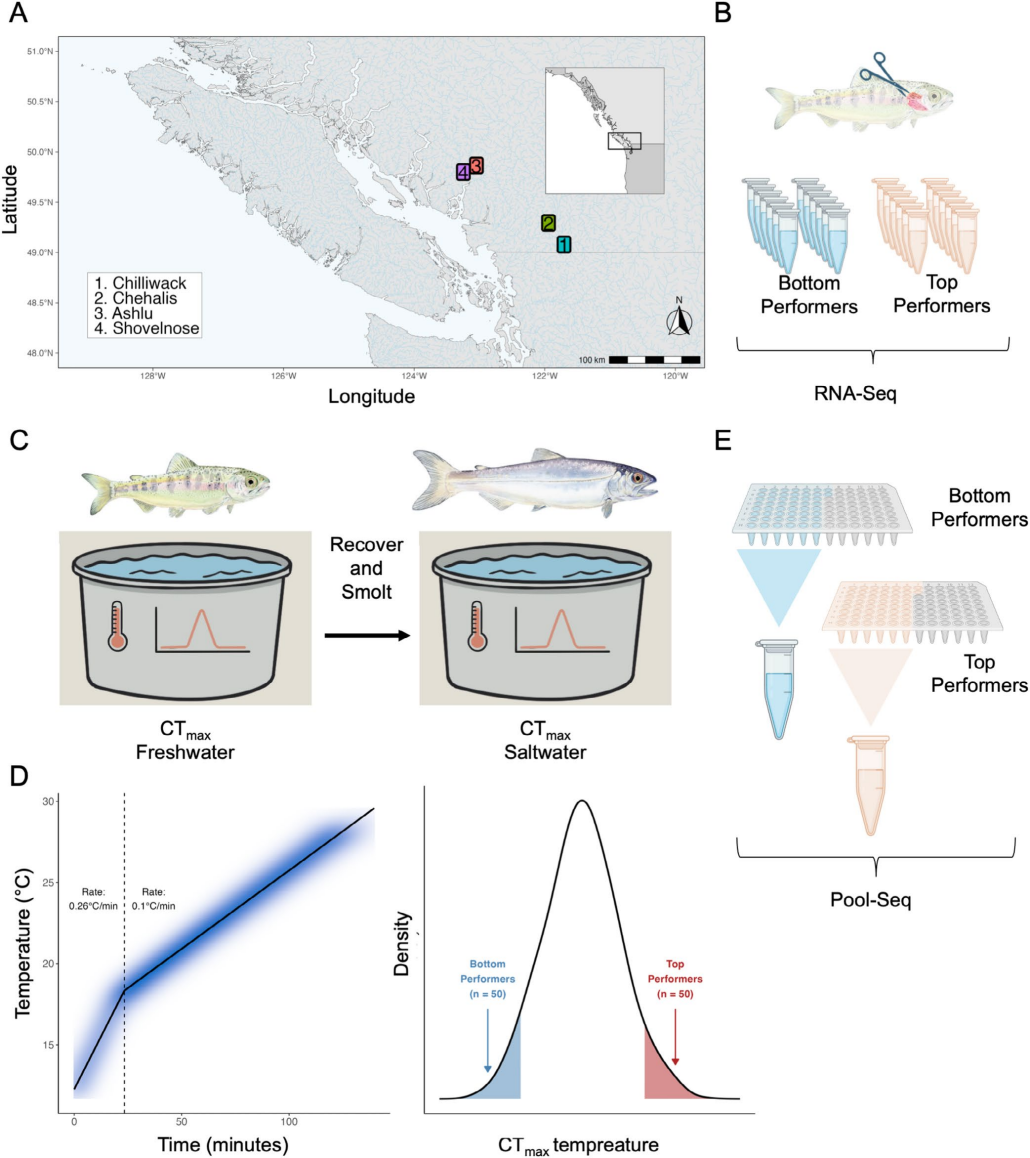
Overview of Chinook salmon populations and experimental design. This figure summarises the geographic distribution of study populations and the experimental design used to assess thermal tolerance in juvenile Chinook salmon. (A) Map of Vancouver Island and the southwest coast of British Columbia showing the locations of the four focal populations, labelled 1–4: 1 = Chilliwack, 2 = Chehalis, 3 = Ashlu, and 4 = Shovelnose. An inset map of the broader Pacific Northwest highlights the study region in a boxed area. (B) Prior to CT_max_ trials, nonlethal gill biopsies were collected for transcriptomic analysis. (C) A repeated‐measures design was used to assess thermal tolerance before and after smoltification within individuals. (D) Temperature ramping profiles during CT_max_ trials from submerged temperature loggers. The black line represents the average ramping rate, while the blue shading shows the full range of individual trial data. The adjacent panel illustrates the CT_max_ distribution, with the top and bottom 5% of individuals marked as high and low performers. (E) DNA from the 50 highest‐ and 50 lowest‐performing individuals per population was pooled for whole‐genome sequencing and allele frequency analysis using PoolSeq.

Approximately 2000 juvenile chinook salmon parr, 500 from each of four hatchery populations, were obtained from DFO hatcheries several weeks before their expected smoltification and were transported to the DFO Pacific Science Enterprise Centre (PSEC) in West Vancouver. The four populations included two stream‐type and two ocean‐type strains (Figure 1). These populations were selected such that all fish would be ready to transition to saltwater at approximately the same time of year, although individuals of the two life‐history types were of different ages at the time of smoltification. Although run timing (e.g., summer vs. fall) often correlates with juvenile life history, these traits can vary independently among Chinook populations. Accordingly, we refer primarily to life‐history type (stream vs. ocean) throughout the manuscript. The stream‐type strains (hereafter, *Ashlu* and *Shovelnose*) are summer‐run populations originating from different tributaries of the Squamish River and are reared at the DFO Tenderfoot Creek Hatchery (Squamish, BC). The ocean‐type strains were sourced from tributaries of the lower Fraser River: *Chehalis* (fall‐run, ocean‐type) from the DFO Chehalis River Hatchery (Agassiz, BC) and *Chilliwack* (summer‐run, ocean‐type) from the DFO Chilliwack River Hatchery (Chilliwack, BC). The Chilliwack strain was originally established in the 1970s through transplantation of summer‐run Chinook salmon from middle‐ and upper‐Fraser tributaries. While some of the founding populations may have exhibited stream‐type traits, the Chilliwack program has been managed for over 50 years as an ocean‐type hatchery stock, with consistent rearing and release as sub‐yearlings. The population exhibits ocean‐type migratory timing and is ecologically classified as such. DFO hatcheries generally operate as genetically integrated programs, where naturally spawned fish from the local watershed are incorporated into broodstock to maintain gene flow between hatchery and wild fish, reduce domestication and preserve genetic diversity. The Chilliwack program currently follows this model, using local returns for broodstock collection.

Animals were reared in compliance with the Canadian Council on Animal Care guidelines, and all live animal procedures were approved by the Animal Care Committees of both Fisheries and Oceans Canada Pacific Region and the University of British Columbia (AUP: A23‐0051). Chinook salmon were housed in aerated fresh well water in 3700 L tanks and fed to satiation daily with size‐appropriate commercial salmon feed (BioClark's Fry; Skretting Canada Ltd., Vancouver, BC). The fish were acclimated to freshwater laboratory conditions for 3 weeks before being anaesthetised (80 mg/L Syncaine buffered with 160 mg/L sodium bicarbonate; Syndel Canada, Nanaimo, BC), PIT‐tagged and nonlethal gill biopsies and fin clips were collected. Gill tissue was stored in RNAlater for subsequent RNA isolation and gene expression analysis, while fin clips were preserved in ethanol for DNA isolation and sequencing (Figure 1). The fish were allowed to recover for 2 weeks prior to the freshwater CT_max_ trial (as described below). Following CT_max_, the fish were allowed to recover for a minimum of 2 weeks prior to transition to saltwater. Fish were determined to be ready for smoltification by the loss of parr marks (dark pigmented spots on their sides) and the development of silver coloration. Fish were transitioned to filtered, UV‐treated saltwater drawn from Burrard Inlet (~29 ppt salinity) over the period of 1 week with an increase of 3 ppt salinity per day. Two weeks following reaching full salinity, the fish were assayed for saltwater CT_max_ (as described below).

### 2.2 Thermal Tolerance (CT_max_ ) Assessment

All fish underwent a CT_max_ trial as a nonlethal measure of acute thermal tolerance by determining the temperature at which they could no longer maintain equilibrium during a thermal ramp. This method is widely used to assess acute thermal tolerance in fish and this trait has been linked to species biogeographic boundaries and sensitivity to climate change (Dahlke et al. 2020; Sunday et al. 2014). We used the method outlined by (Strowbridge et al. 2021). Briefly, the fish were held off feed for 24 h prior to the trial. Fish were assayed for CT_max_ in batches of approximately 100 fish (range: 77–106). All trials started between 9 and 11 am. Fish were netted from the home tank and transferred to a 400 L tank containing the same water source as their home tank. They were allowed to acclimate to the test tank for 30 min. Each CT_max_ trial started with an initial average temperature ramp rate of 0.26°C/min until the trial tank reached 18°C, at which point the temperature ramp rate was reduced to 0.1°C/min for the remainder of the trial (Figure 1). At least four people were involved in each trial: one to monitor and record the temperature, two to observe the tank and remove fish as they lost equilibrium, and one to scan PIT tags. Once fish lost equilibrium they were immediately netted, had their PIT tag scanned, and were returned to a recovery tank containing water at ambient temperature.

To evaluate differences in thermal tolerance across populations and salinity treatments, we used a linear mixed‐effects model (LMM) with population, water type (freshwater or saltwater) and their interaction as fixed effects, and trial as a random effect to account for experimental batch variation. The model was fitted using the lmer() function from the lme4 package (v 1.1.37) (Bates et al. 2015), and the statistical significance of fixed effects was assessed using Satterthwaite's method via the lmerTest package (v3.1.3) (Kuznetsova et al. 2017). Post hoc pairwise comparisons among all population‐by‐salinity groups were conducted using the emmeans package (v1.11.0), with Tukey's adjustment for multiple testing.

### 2.3 DNA Sequencing and Genetic Analysis

DNA was extracted from tail fin clip tissue using a Qiagen DNeasy Blood and Tissue Kit, following the manufacturer's recommended protocol. DNA concentration was measured using a Quant‐iT PicoGreen dsDNA Assay. Genetic sex of Chinook salmon was determined using a TaqMan quantitative PCR (qPCR) assay targeting the male‐specific GHY locus, with the insulin‐like growth factor 1 (IGF1) gene used as an internal positive control (Balbag et al. 2019). Each 10 μL reaction contained 5 μL of TaqMan Fast Advanced Master Mix (Applied Biosystems), 0.6 μL each of GHY1‐F (5′‐GATGACAATGACTCTCAGCATCTG‐3′) and GHY2‐R (5′‐GACCCAAAGATACGTCCAGGTT‐3′) primers, 0.15 μL of GHY‐FAM probe (5′‐ATGCGGGAACTAATG‐3′), 0.3 μL each of IGF1‐F (5′‐TGCGATGTGCTGTATCTCCTGTA‐3′) and IGF1‐R (5′‐CCTGTTGCCGCCGAAGT‐3′) primers, and 0.1 μL of IGF1‐VIC probe (5′‐TCTCACTGCTGCTGTGC‐3′). Reactions were run on a Bio‐Rad CFX Opus 96 Real‐Time PCR System using the following cycling protocol: 95°C for 20 s, followed by 40 cycles of 95°C for 3 s and 60°C for 30 s. Fluorescence from FAM and VIC dyes was monitored to detect amplification of GHY and IGF1, respectively. Samples with earlier or strong GHY amplification relative to IGF1 were classified as genetic males. Samples showing only IGF1 amplification, or delayed/absent GHY signal, were classified as genetic females.

For each population, genomic DNA was pooled from the 25 males and 25 females with the highest CT_max_ values to create the ‘top performer’ sample, and from the 25 males and 25 females with the lowest CT_max_ values to create the ‘bottom performer’ sample, ensuring each pool contained 50 individuals with a balanced sex ratio. Pooled DNA samples were submitted to the Génome Québec Innovation Centre for library preparation and high‐throughput sequencing on the Illumina HiSeq X platform (paired‐end 150 bp reads). Each library was distributed across three sequencing lanes to ensure even coverage and subsequently merged for downstream analysis. Each DNA pool was sequenced to an average depth of 86X (904,920,819 reads ± 152,986,984 SD). Poolseq adapters were trimmed, and reads were quality filtered using TrimGalore! The filtered reads were mapped to the Chinook salmon genome assembly (NCBI assembly GCF_018296145.1_Otsh_v2.0) using BWA.

Genotype analysis followed the PoPoolation2 pipeline (Kofler et al. 2011) to process and analyse allele frequencies and genetic differentiation between top and bottom performers. Low‐quality reads were filtered by applying a mapping quality threshold of 20 using samtools view ‐q 20 ‐bS, followed by sorting with samtools sort. Synchronised files containing allele frequencies for each group were generated with samtools mpileup ‐B and converted to the synchronised format using mpileup2sync.pl with a minimum quality threshold of 20. Allele frequency differences between performance groups were calculated using snp‐frequency‐diff.pl, with a minimum count of 6 and coverage between 50 and 200. Fst values were computed using fst‐sliding.pl with a sliding window approach (window sizes of 1 or 500 bases), applying a minimum coverage of 50, maximum coverage of 200, and the ‐‐suppress‐noninformative option to exclude non‐SNP windows.

To detect consistent allele frequency changes associated with thermal tolerance, a Cochran–Mantel–Haenszel (CMH) test was applied using the top and bottom performers from different populations as biological replicates. The CMH test was performed on these replicated allele frequencies using the cmh‐test.pl script, comparing allele frequencies between populations with a minimum count of 12 and coverage between 50 and 200. In addition, to identify population‐specific signals of thermal tolerance, Fisher's exact tests were performed separately for each population using the fisher‐test.pl script from the PoPoolation2 pipeline. This test compared allele frequencies between top and bottom performers within each population, applying the same filtering thresholds for minimum count and coverage.

To assess the functional relevance of genes associated with significant SNPs, Gene Ontology (GO) enrichment analyses were performed using the *goseq* package (Young et al. 2010). SNPs were filtered at a *p* value threshold of 1 × 10^−4^, and adjusted *p* values were calculated using the Benjamini–Hochberg correction to account for multiple testing. Genomic coordinates of significant SNPs were expanded into ±100 kb windows to identify nearby genes, capturing potential cis‐regulatory regions and linkage blocks. For each population, overlapping genes were extracted, and GO enrichment was conducted with *goseq*. GO annotations were obtained from NCBI for the Chinook salmon genome assembly, and categories containing fewer than 10 genes were excluded to minimise inflation of enrichment statistics. Enrichment was tested for biological process (BP) terms, with significance defined as *p* < 0.05 after Benjamini–Hochberg adjustment.

### 2.4 RNA Sequencing and Gene Expression Analysis

Non‐lethal gill clips were taken from each individual approximately 3 weeks before the first CT_max_ trial and smoltification and preserved in RNAlater. Total RNA was extracted from tissue samples using the TRIzol reagent (Invitrogen) following the manufacturer's protocol. Briefly, tissues were homogenised in 1 mL of TRIzol reagent, and 200 μL of chloroform was added to facilitate phase separation. The aqueous phase containing RNA was collected after centrifugation, and RNA was precipitated with equal volume of isopropanol. The RNA pellet was washed with 1 mL of 75% ethanol, air‐dried and resuspended in nuclease‐free water. To remove any contaminating genomic DNA, an on‐column DNase treatment was performed using the Qiagen RNase‐Free DNase Set, following the manufacturer's instructions. DNase treated total RNA samples were shipped to the Genome Quebec sequencing facility on dry ice for subsequent library preparation and sequencing using the Illumina HiSeq X PE150 platform. A total of 67,377,532 ± 7,257,510 SD reads were generated from each sample. Adapters were trimmed and reads were quality filtered using trimmomatic. Reads were aligned to the Chinook salmon genome assembly (NCBI assembly GCF_018296145.1_Otsh_v2.0) using STAR (Dobin et al. 2013).

Gene‐level read counts were quantified and analysed using the edgeR package (Robinson et al. 2010). Genes with low expression were filtered by retaining only those with counts per million (CPM) > 0.5 in at least 70% of individuals per group. Data were normalised using the trimmed mean of *M*‐values (TMM) method. For each population, a generalised linear model (GLM) was used to compare gene expression between individuals classified as high vs. low performers based on CT_max_. Differential expression was assessed using a quasi‐likelihood *F*‐test (glmQLFTest), with significance defined as FDR < 0.1 (Benjamini–Hochberg correction). Principal component analysis (PCA) was conducted on log‐transformed CPM values to visualise patterns of transcriptomic variation and assess separation between performance groups within each population.

To investigate the functional relevance of differentially expressed genes (DEGs), gene ontology (GO) enrichment analysis was conducted using the goseq package (Young et al. 2010), with expressed genes used as the background and gene length bias accounted for. GO annotations were obtained from NCBI for the Chinook salmon genome assembly. Terms with FDR‐adjusted *p*‐values < 0.05 were considered enriched. In populations lacking significant GO enrichment, we applied an interquartile range (IQR) overlap filter to identify DEGs with consistent expression differences between performance groups, focusing on those with minimal overlap in the central 50% of expression values (25th–75th percentile).

## 3 Results

### 3.1 Population and Life‐Stage Variation in Thermal Tolerance

Sex‐specific differences in thermal tolerance are variable across fish species, with some studies reporting no difference between males and females and others observing slight or context‐dependent differences (Bartlett et al. 2022; Morgan et al. 2018; O'Donnell et al. 2020; Vallin et al. 2025; Yanar et al. 2023). We compared CT_max_ between males and females and found no significant differences in any population, or in freshwater or saltwater (Figure S1). Consequently, sexes were pooled for all subsequent analyses.

**FIGURE 2:**
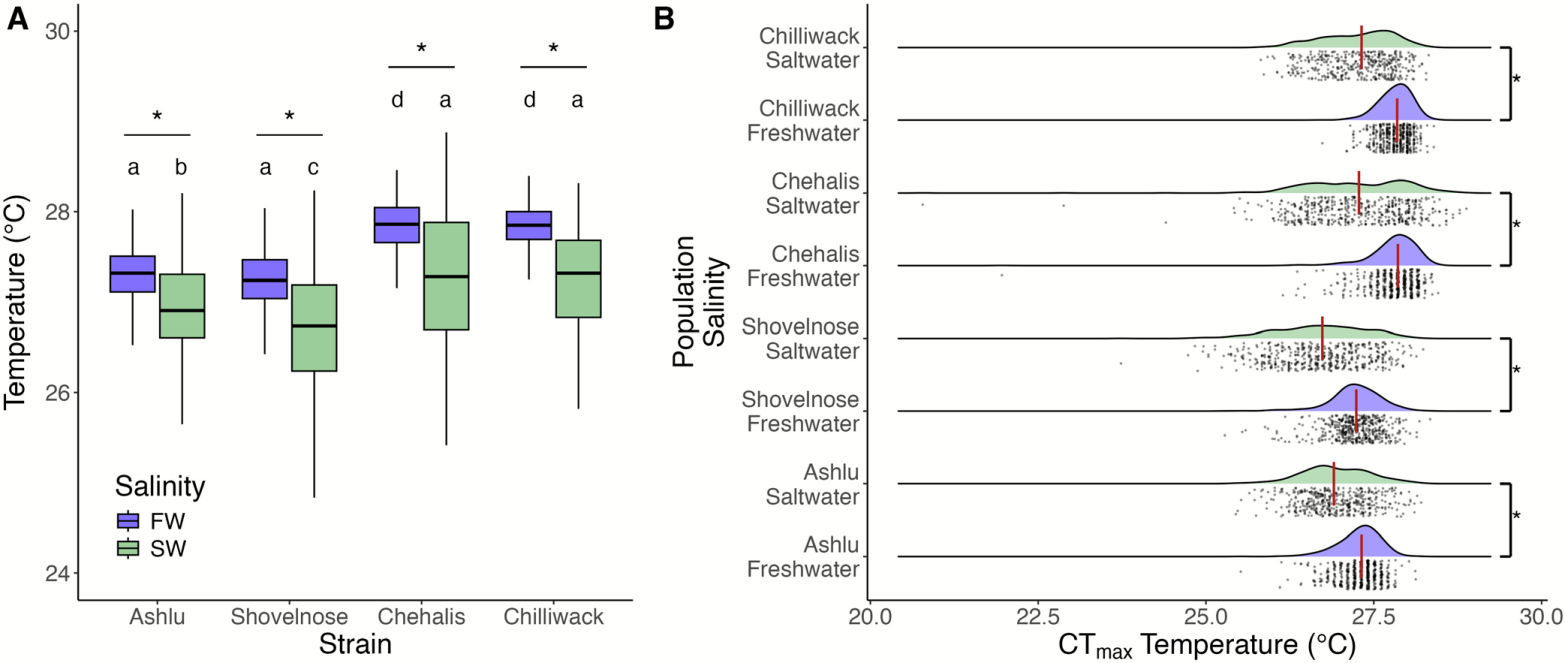
Effects of life history strategies and smoltification on thermal tolerance in Chinook salmon. (A) Boxplots of critical thermal maxima (CT_max_) for four populations of juvenile Chinook salmon ( *Oncorhynchus tshawytscha* ) sampled as pre‐smolts in freshwater (FW, light blue) and postsmoltification in full‐strength saltwater (SW, green). Lowercase letters indicate statistically significant differences among populations in freshwater; uppercase letters indicate differences among populations in saltwater. Asterisks denote significant within‐population differences between freshwater and saltwater treatments (*p* < 0.05). (B) Raincloud plots showing the distribution of CT_max_ values by population and salinity treatment. Kernel density ridges are overlaid with individual observations and median lines (red). Thermal tolerance is reduced after smoltification, with individuals in saltwater displaying more variation in their thermal tolerance compared to those in freshwater. Brackets and asterisks indicate significant effects of smoltification on CT_max_ within each population.

We hypothesised that stream‐type Chinook salmon, due to their extended residency time in freshwater and increased probability of exposure to temperature extremes, would exhibit higher thermal tolerance compared to ocean‐type populations. However, contrary to this prediction, we found that the CT_max_ of stream‐type Chinook salmon was significantly lower than that of ocean‐type salmon in both freshwater (presmolt) and saltwater (post‐smolt) conditions (*p* < 0.05) (Figure 2). Across all populations, smoltification was associated with a significant reduction in CT_max_ (−0.35°C, *p* < 0.001), suggesting that the physiological changes associated with this process negatively impact thermal tolerance. Moreover, the magnitude of this reduction varied across populations, ranging from 0.35°C in the stream‐type Ashlu population to larger declines in Shovelnose (0.52°C) and the two ocean‐type populations (0.54°C in Chehalis, 0.57°C in Chilliwack). Summary statistics of CT_max_ values can be found in Tables S1–S3. We also noted lower variation in CT_max_ among individuals in freshwater compared to saltwater, indicating that salinity may influence the degree of interindividual variation in thermal tolerance.

**FIGURE 3:**
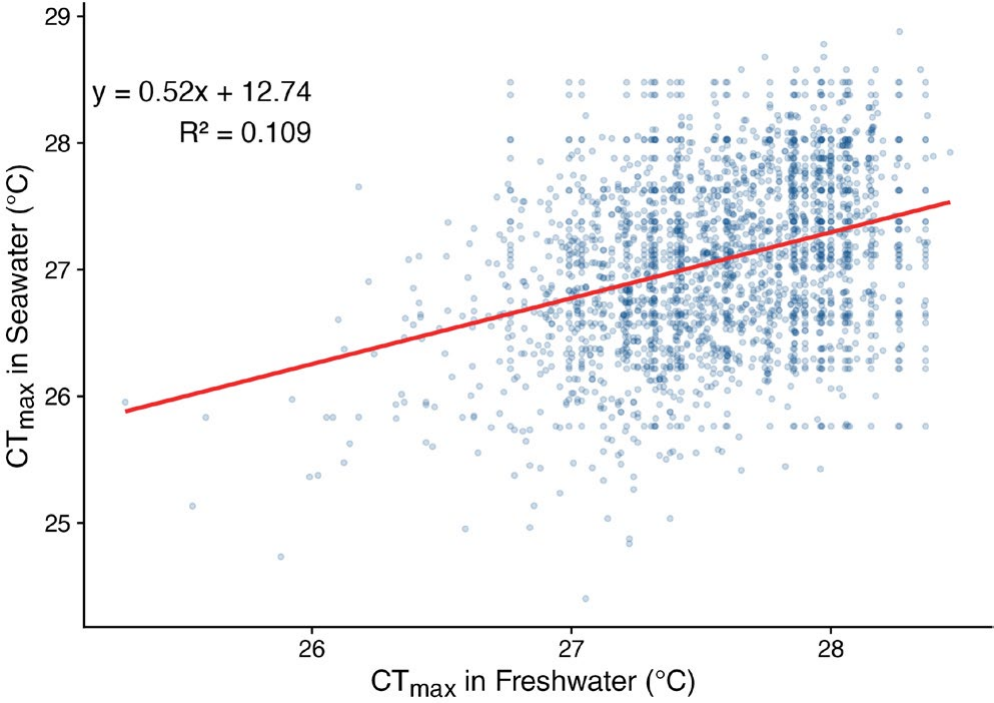
Relationship between CT_max_ in freshwater (FW) and saltwater (SW). Scatter plot depicting CT_max_ values measured in both FW and SW for the same individuals, with a linear regression line (red) illustrating the relationship. The regression equation and *R* ^2^ value are shown on the plot. A single outlier point was omitted for clarity.

Because individuals were PIT‐tagged and tracked across both stages, we were able to compare CT_max_ within individuals before and after smoltification to assess whether thermal tolerance in freshwater predicted tolerance in saltwater. One outlier was excluded from this analysis for clarity (full model shown in Figure S2). We found only a weak correlation between pre‐ and postsmolt CT_max_ (*R* ^2^ = 0.109) (Figure 3).

We note that stream‐type Chinook salmon were 1 year older than ocean‐type fish and therefore larger; summary statistics for weight, fork length and condition factor for each population are provided in Table S4. Because CT_max_ comparisons and selection of top‐ and bottom‐performing individuals for sequencing were conducted within the same cohort and age class for each population, these size differences do not influence classification of thermal performance within a population.

### 3.2 Genome‐Wide Association of Genetic Variants Underlying Thermal Performance

To identify genetic variants associated with thermal tolerance, we pooled DNA from the top and bottom 50 individuals per population based on CT_max_, aiming for a 1:1 sex ratio (25 males, 25 females per pool). This approach was intended to minimise sex‐linked genetic artefacts, particularly those arising from differentiation on the sex chromosome. The CT_max_ values used to rank individuals were those measured during the freshwater thermal tolerance trials, enabling direct comparison with transcriptomic data from the same individuals. Fish weight (WT) and fork length (FL) did not statistically differ between top and bottom performers in any population, and pools were comparable in size and condition (Shovelnose: WT top = 22.5 ± 5.85 g, bottom = 21.3 ± 5.40 g, *p* = 0.25; FL top = 12.29 ± 1.01 cm, bottom = 12.15 ± 0.97 cm, *p* = 0.47; Chehalis: WT top = 7.64 ± 1.38 g, bottom = 8.13 ± 1.88 g, *p* = 0.14; FL top = 8.70 ± 0.54 cm, bottom = 8.83 ± 0.64 cm, *p* = 0.28; Chilliwack: WT top = 11.09 ± 2.00 g, bottom = 10.58 ± 1.61 g, *p* = 0.17; FL top = 9.90 ± 0.57 cm, bottom = 9.79 ± 0.47 cm, *p* = 0.32; Ashlu: WT top = 17.11 ± 3.24 g, bottom = 17.41 ± 3.13 g, *p* = 0.64; FL top = 11.61 ± 0.66 cm, bottom = 11.69 ± 0.60 cm, *p* = 0.51).

**FIGURE 4:**
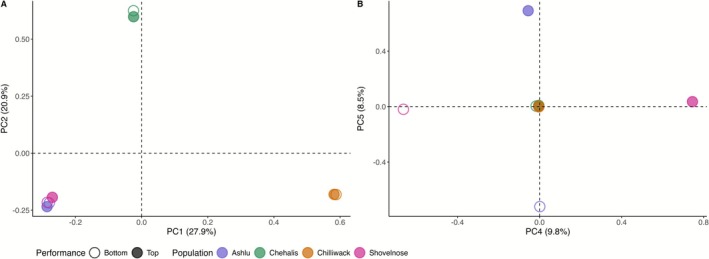
Principal component analysis (PCA) of allele frequency variation associated with thermal tolerance in four populations of Chinook salmon. (A) PC1 and PC2 separate individuals by population, highlighting population‐level structure. (B) PC4 and PC5 distinguish top‐ and bottom‐performing individuals within the stream‐type life history, reflecting variation associated with thermal performance. Each point represents a pooled sequencing sample comprising individuals from the same population and performance group, with colours indicating performance (Top vs. Bottom) and shapes denoting population of origin. Populations are distinguished by colour: Ashlu (slate blue), Chehalis (green), Chilliwack (orange) and Shovelnose (magenta). Performance groups are distinguished by shape: Filled symbols indicate top performers and open symbols indicate bottom performers.

Principal component analysis (PCA) of the Poolseq data revealed clear genetic differentiation among populations: The first two principal components explained 48.8% of the total variance and primarily separated individuals by population and life‐history type, indicating that interpopulation divergence is stronger than genome‐wide signals associated with thermal tolerance (Figures 4, S3). Notably, differentiation between top and bottom CT_max_ performers became evident in PC4 (9.8%) and PC5 (8.5%), particularly within the two stream‐type populations (Shovelnose and Ashlu).

**FIGURE 5:**
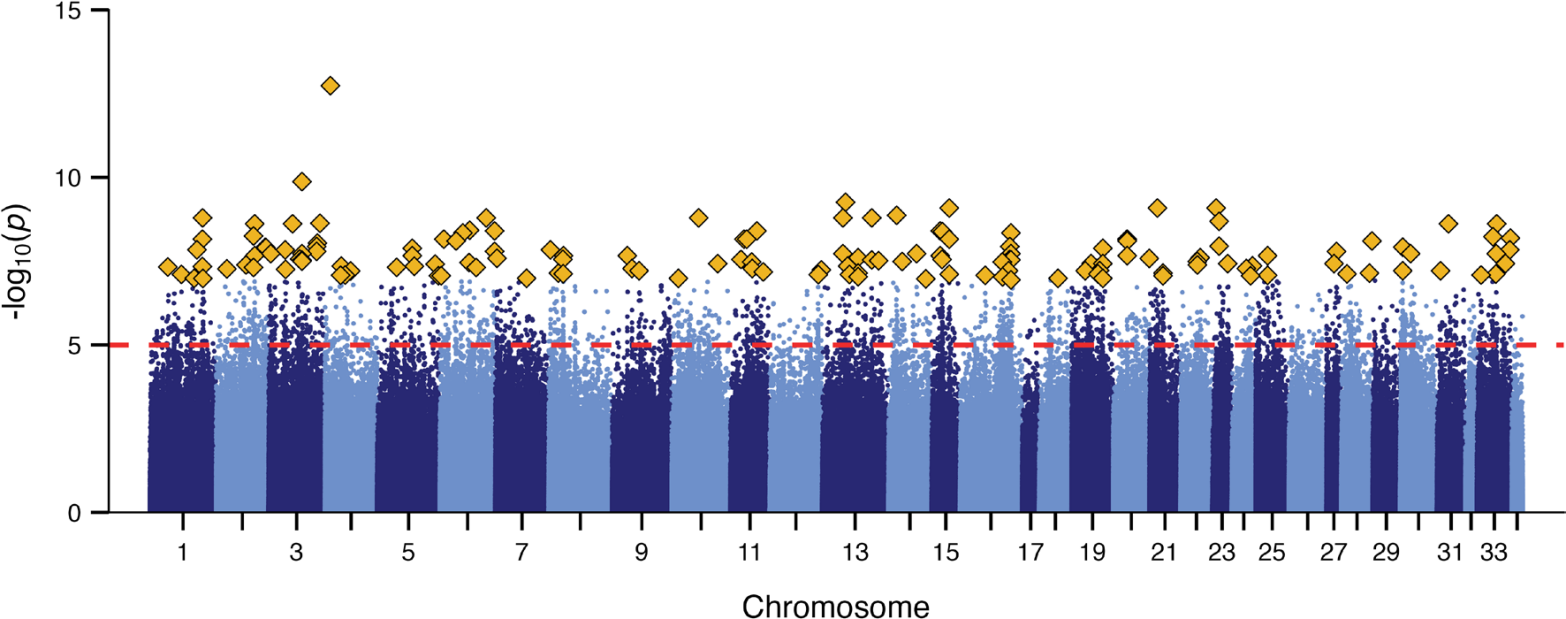
Manhattan plot of allele frequency variation associated with thermal tolerance across the Chinook salmon genome. Each point represents a single SNP, plotted by genomic position (*x*‐axis) and statistical significance (*y*‐axis; −log_10_ transformed FDR‐adjusted *p* value). Alternating colours distinguish different chromosomes. The dashed red line indicates the genome‐wide significance threshold (FDR < 1e–5). The top 150 most significant SNPs are highlighted as gold diamonds. SNP frequencies were estimated from poolseq data, where each pool comprised individuals with either the 50 highest or 50 lowest CT_max_ temperatures in freshwater from each of four Chinook salmon populations. These loci represent the strongest associations with variation in thermal tolerance.

To identify loci associated with thermal tolerance across populations, we applied the Cochran–Mantel–Haenszel (CMH) test to detect consistent allele frequency differences between high and low performers. This analysis identified 2454 SNPs with *p* values < 1 × 10^−8^, including 453 SNPs with *p* values < 1 × 10^−10^. These SNPs were broadly distributed across the genome, with no evidence of clustering on specific chromosomes or regions (Figure 5). The SNP with the lowest *p* value was located downstream of LOC112246624 on chromosome 4, near *dnajc*, a member of the Hsp40 (DNAJ) family known to play a key role in cellular responses to thermal stress. A list of the genes associated with the top 20 SNPs with the lowest *p*‐values is provided in Table 1. To investigate the biological processes associated with thermal tolerance, we performed Gene Ontology (GO) enrichment analysis on genes located near significant SNPs. This analysis identified two enriched processes: the IRE1‐mediated unfolded protein response and the intrinsic apoptotic signalling pathway in response to endoplasmic reticulum stress. Both processes are involved in the cellular stress response triggered by the accumulation of unfolded proteins in the endoplasmic reticulum and are consistent with pathways expected to be under selection for enhanced thermal tolerance.

**TABLE 1:** Gene associations for SNPs linked to thermal tolerance in Chinook salmon.

SNP	chr	Position	*p*	Genes
SNP1	NC_056432.1	7,781,624	6.75^e‐20^	LOC121846410, LOC112246624
SNP2	NC_056431.1	48,311,998	9.85^e‐17^	LOC112237713, rab3gap1, LOC112246698, LOC112246715, LOC112246716, LOC112246703
SNP3	NC_056441.1	33,790,334	6.10^e‐16^	LOC112264749, LOC112264750
SNP4	NC_056449.1	11,414,335	1.42^e‐15^	LOC112220940
SNP5	NC_056443.1	25,417,071	1.70^e‐15^	LOC112214886, LOC112214707, LOC112214705, LOC112214708, LOC112214709, LOC112214710, LOC112214711, aco1, LOC112214713
SNP6	NC_056451.1	5,048,761	1.81^e‐15^	LOC112238818, LOC112236444, LOC112236443, LOC112236446, LOC112236445, LOC112236442, LOC121840578, LOC112236447, ubxn11, LOC121840648, si:ch211‐154o6.3, LOC121840579
SNP7	NC_056442.1	11,986,235	3.49^e‐15^	LOC112255179, LOC112267731, si:ch211‐246m6.5, LOC112267733, LOC112267734
SNP8	NC_056438.1	38,738,316	5.08^e‐15^	efr3a, LOC112250070
SNP9	NC_056441.1	71,685,159	5.97^e‐15^	LOC112265640, LOC112265976, LOC112265642, LOC112265641, wsb1
SNP10	NC_056441.1	30,017,094	6.88^e‐15^	LOC112264662, LOC112264663, LOC112266005, syngap1a
SNP11	NC_056429.1	75,999,504	7.05^e‐15^	LOC121847482, LOC112256887, LOC112256896, LOC112256913, LOC112256921, LOC121846908
SNP12	NC_056434.1	66,974,294	7.15^e‐15^	nt5c2a, inaa, pcgf6, LOC112252945, calhm2.1, LOC112253522, LOC112252946, LOC112252947, LOC112253417, LOC112252948, LOC121846720, LOC112253523, LOC112252949
SNP13	NC_056451.1	9,369,064	9.82^e‐15^	LOC112230421, lsr, LOC112230403, LOC112223692, si:dkey‐199f5.8, psmc4, scn1ba, gramd1a
SNP14	NC_056430.1	55,451,130	1.38^e‐14^	ergic3, romo1, mych, mtss1
SNP15	NC_056431.1	34,695,053	1.58^e‐14^	LOC112234792, LOC112224044
SNP16	NC_056461.1	28,436,712	1.61^e‐14^	LOC121841700, LOC112231074, LOC112231075, LOC112231076, LOC112231078, slc2a11l
SNP17	NC_056459.1	16,644,041	1.61^e‐14^	LOC112229625
SNP18	NC_056434.1	43,059,693	2.65^e‐14^	tmtc2b
SNP19	NC_056443.1	12,420,270	3.00^e‐14^	LOC112214425, LOC112214426
SNP20	NC_056439.1	38, 026, 378	3.11^e‐14^	LOC112261964, LOC112261881, LOC112261880, LOC112262193

We next applied Fisher's exact tests to compare allele frequencies between top and bottom performers within each population. A greater number of significant SNPs were identified in the stream‐type populations (2502 in Ashlu; 18,090 in Shovelnose) compared to the ocean‐type populations (322 in Chehalis; 90 in Chilliwack) (Figure S4). No SNPs were consistently associated with performance across all four populations (Figure S5). These results are consistent with the patterns observed in the PCA, where separation between performance groups was more pronounced in the stream‐type populations.

Gene ontology enrichment analysis of genes near significant SNPs at the individual population level identified distinct sets of biological processes associated with thermal tolerance across Chinook salmon populations. While each population exhibited a unique profile of enriched terms (see Tables S5–S8), some common themes were evident. Both ocean‐type populations (Chilliwack and Chehalis) showed enrichment for signalling and transport processes, including cytokine production, the calcineurin‐NFAT signalling cascade and retrograde vesicle transport. In contrast, stream‐type populations (Ashlu and Shovelnose) showed enrichment for processes related to transcription, translation and cellular stress responses, including apoptotic signalling in response to ER stress, synaptic function, oxidative stress response and the unfolded protein response. Stress‐related pathways were enriched in both life histories, although stream‐type populations exhibited a broader diversity of additional processes linked to neuronal signalling and structural organisation. These patterns suggest population‐specific strategies for maintaining cellular function under thermal stress.

### 3.3 RNA‐Seq Analysis of Thermal Performance in Chinook Salmon

**FIGURE 6:**
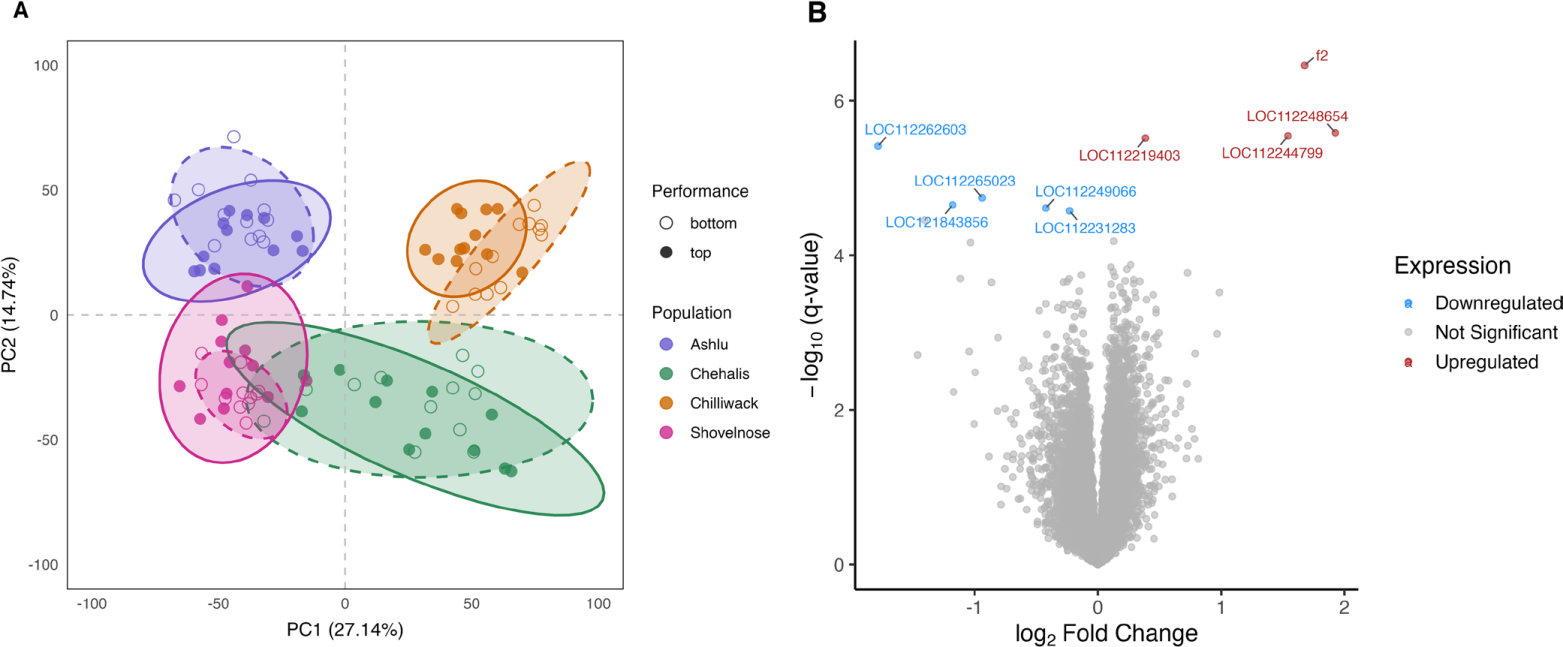
Transcriptomic patterns associated with thermal tolerance in Chinook salmon. (A) Principal component analysis (PCA) of RNA‐seq data from four Chinook salmon populations, separated by thermal tolerance performance groups (top and bottom performers based on CT_max_). PCA was performed on normalised counts per million, with PC1 and PC2 explaining 27.14% and 14.74% of the variance, respectively. Points represent individual samples, coloured by population: Ashlu (blue), Chehalis (green), Chilliwack (orange), and Shovelnose (pink). Individuals are grouped by thermal performance based on their critical thermal maximum (CT_max_): Top performers (filled circles) have higher CT_max_ temperatures, while bottom performers (open circles) have lower CT_max_. Ellipses represent 95% confidence intervals for each group, with solid lines for top performers and dashed lines for bottom performers. (B) Volcano plot showing differential gene expression between top and bottom performers across all populations. The *x*‐axis represents log_10_ fold change in expression (top vs. bottom performers), while the *y*‐axis shows −log_10_ of the FDR‐adjusted *p* value (*q*‐value). Genes with FDR < 0.1 are coloured by direction of change: Upregulated (red), downregulated (blue), and not significant (grey). All significantly differentially expressed genes (DEGs) are labelled. The plot highlights modest transcriptional differences associated with thermal performance, including both up‐ and downregulated genes.

Principal component analysis (PCA) of the RNA‐seq data revealed that samples primarily clustered by population and life‐history type, with little separation between top and bottom thermal performers. PC1 and PC2 together accounted for 41.88% of the total variance (27.14% and 14.74%, respectively) (Figure 6). These results suggest that population‐level differences in transcript abundance are the primary source of variation in the data set, with performance‐related expression differences being comparatively subtle.

Differential expression analysis including all populations identified nine genes with significant expression differences between top and bottom performers. However, manual inspection showed that mean expression differences were often driven by a small number of outlier samples.

To mitigate the influence of outliers, we compared the interquartile range (IQR) of gene expression between groups and ranked genes based on the degree of IQR overlap. Three genes (STARD7, ZNF711 and TNFRSF3 (Table S9)) exhibited less than 20% IQR overlap between top and bottom performers, suggesting consistent expression differences across most individuals. While these genes showed modest log‐fold changes, their expression patterns were more robust and less influenced by outliers, making them the strongest candidates for association with thermal performance.

**FIGURE 7:**
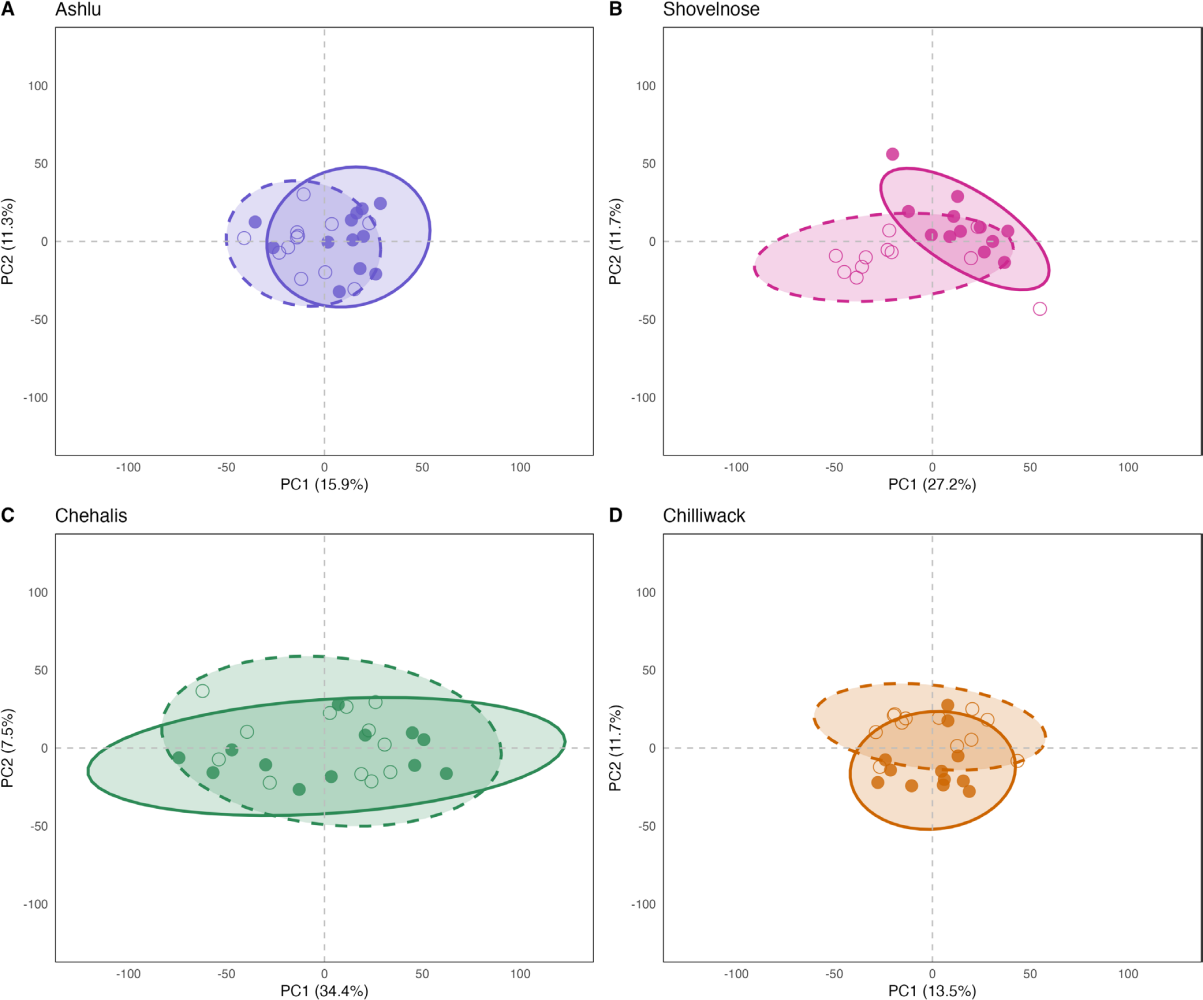
Principal component analysis (PCA) of gene expression variation across four Chinook salmon populations. Panels A–D show PC1 vs. PC2 plots for the Ashlu, Shovelnose, Chehalis, and Chilliwack populations, respectively. Each point represents an individual, coloured by population (Ashlu = blue, Shovelnose = pink, Chehalis = green, Chilliwack = orange). Individuals are grouped by thermal performance based on their critical thermal maximum (CT_max_): top performers (filled circles) have higher CT_max_ temperatures, while bottom performers (open circles) have lower CT_max_. Ellipses represent 95% confidence intervals for each group, with solid lines for top performers and dashed lines for bottom performers. Axis labels indicate the percent variance explained by each principal component.

Given the dominant effect of population on gene expression profiles, we next examined expression differences between top and bottom performers within each population independently. PCA revealed that the strongest separation between performance groups occurred along the first principal component (PC1) in the stream‐type populations. In Shovelnose, top and bottom performers separated along PC1, which explained 27.2% of the variance, while Ashlu exhibited weaker but still visible separation along PC1 (15.9% variance explained). In contrast, the ocean‐type populations showed little to no separation along PC1, with only slight divergence observed in Chilliwack across higher‐order components and no clear separation in Chehalis. These results suggest population‐specific transcriptomic patterns associated with thermal performance, even within the same life‐history type (Figure 7).

**FIGURE 8:**
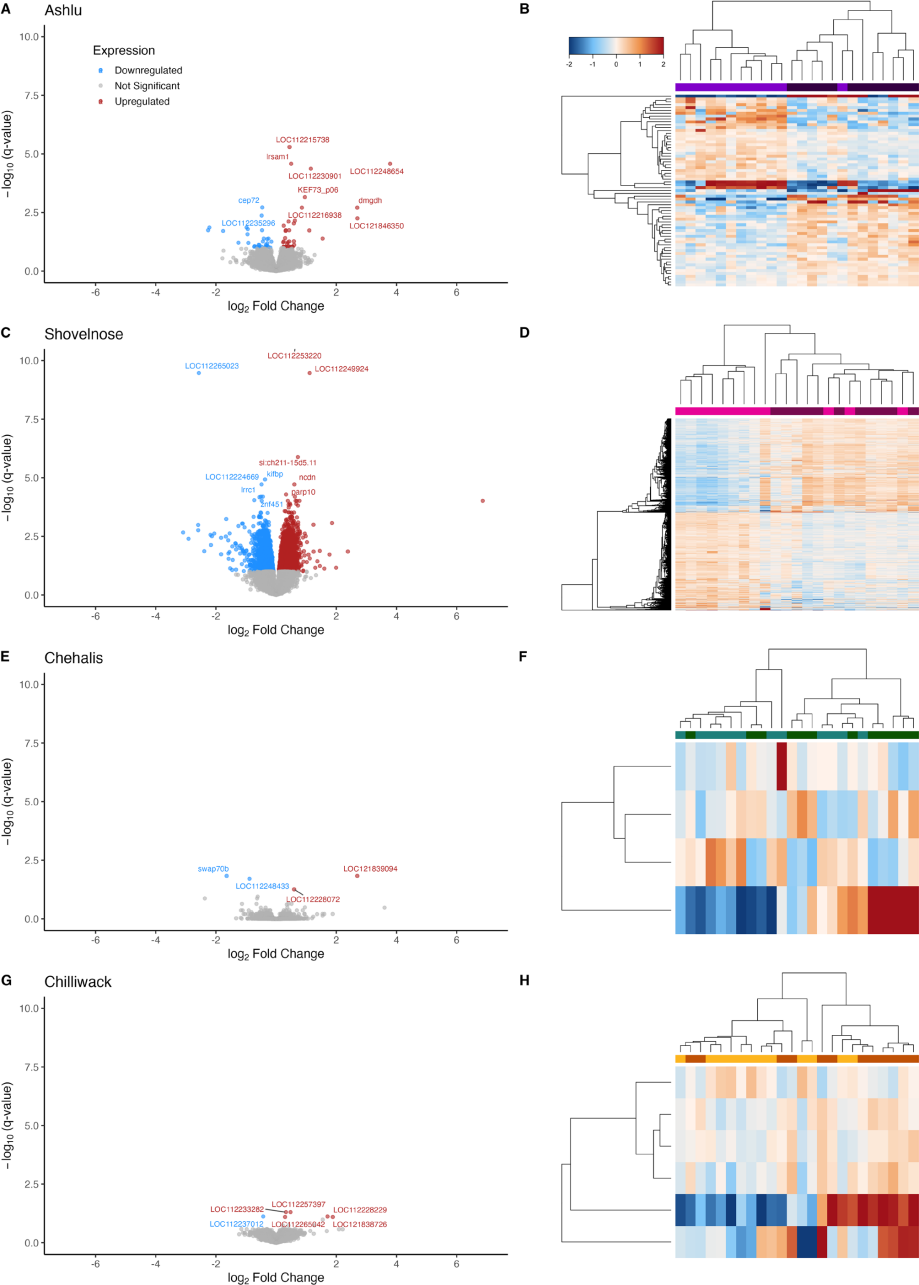
Differential gene expression between top and bottom thermal performers across four Chinook salmon populations. Each row in the figure corresponds to one population: Ashlu (A–B), Shovelnose (C–D), Chehalis (E–F), and Chilliwack (G–H). Left panels (A, C, E, G) show volcano plots comparing gene expression between top and bottom CT_max_ performers within each population. Genes are coloured by significance: Upregulated in top performers (red), downregulated (blue) and not significant (grey). *X*‐axes represent log_2_ fold change; *Y*‐axes represent –log_10_ of the FDR‐adjusted *p* value (*q*‐value). Top significant genes are labelled. Right panels (B, D, F, H) show clustered heatmaps of differentially expressed genes (DEGs) across individuals within each population. Rows represent DEGs, and columns represent individual samples. Expression values for the heatmaps are normalised to the row mean for each DEG. Hierarchical clustering was applied to both genes and individuals, with dendrograms indicating expression similarity. The top dendrogram colour bar distinguishes high (dark) and low (light) thermal performers, highlighting clustering by performance group.

Differential gene expression analysis was consistent with these PCA results. Multiple DEGs were detected in the stream‐type populations (Ashlu: 67 genes; Shovelnose: 6625 genes), where PCA showed clearer separation between top and bottom performers along major principal components. In contrast, the ocean‐type populations, which showed little separation in PCA space, yielded very few DEGs (Chehalis: 4 genes; Chilliwack: 6 genes) (Figure 8).

GO enrichment analysis of DEGs identified several biological processes potentially linked to thermal performance. In Shovelnose, enriched terms included protein ubiquitination and mRNA splicing via the spliceosome (Table S10). In contrast, no significantly enriched GO terms were identified in Ashlu, Chehalis, or Chilliwack. The absence of enrichment in Ashlu may reflect greater heterogeneity in the transcriptional response among individuals or limitations in current functional annotation. Likewise, the limited number of DEGs in Chehalis and Chilliwack likely constrained the ability to detect enriched pathways and aligns with the weaker separation observed in PCA.

To explore potential biological relevance of DEGs in populations without enriched GO terms, we applied an interquartile range (IQR) overlap filter to identify genes with consistent expression differences between performance groups. This approach highlights genes whose central expression ranges (25th–75th percentile) show minimal overlap, reducing the influence of outliers. In Ashlu, this filter identified genes such as *lrsam1*, *IRE1α* (LOC112214707), *dmgdh* and the HMG‐box transcription factor *BBX* (LOC112228428), all of which are associated with stress responses, protein regulation or transcriptional control.

Among the four DEGs in the Chehalis population, only one gene (CIAO1) exhibited an expression pattern where the interquartile ranges of high and low performers showed minimal overlap. Similarly, although six DEGs were identified in the Chilliwack population, most showed substantial overlap in expression distributions. The two genes with the most distinct patterns included one with higher expression in high performers and partial homology to ZNF574, and another (fstl1b; LOC112237012) with lower expression in high performers. While expression differences in ocean‐type populations were relatively modest, the distinct sets of genes implicated across populations further emphasise transcriptomic divergence in thermal performance responses.

### 3.4 SNP Associations With CT_max_ Pre‐ and Postsmolt

**FIGURE 9:**
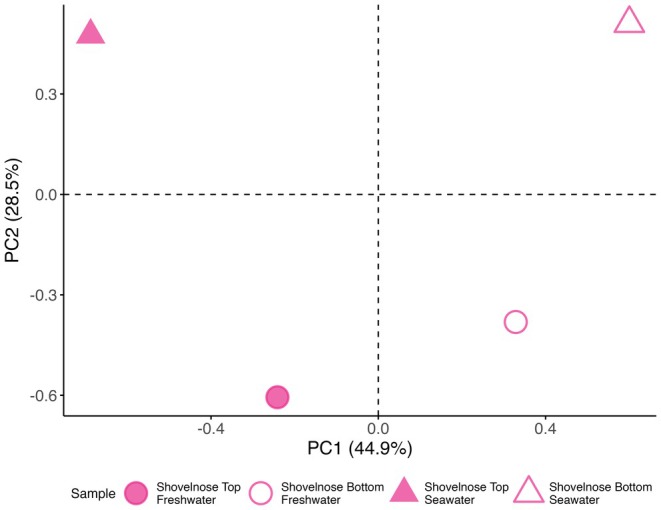
Principal component analysis (PCA) of SNP allele frequencies in Shovelnose Chinook salmon before and after smoltification (freshwater vs. saltwater conditions). Each point represents a pooled sequencing sample comprising individuals from the same salinity and performance group, with shape indicating salinity (circle = freshwater, triangle = saltwater) and fill style representing performance group (filled = top performers, open = bottom performers). Axes represent the first two principal components (PC1 and PC2), which together explain 73.4% of the total variance. Dashed lines denote zero on each axis.

To investigate whether the genomic architecture of thermal tolerance shifts following smoltification, we conducted separate genotype–phenotype association analyses in freshwater and saltwater for the Shovelnose population. Principal component analysis (PCA) of pooled genotypic data from pre‐ and postsmolt samples revealed that the first principal component (PC1), accounting for 44.9% of the total variation, separated pools based on thermal performance, distinguishing top and bottom performers. The second principal component (PC2), explaining 28.5% of the variation, distinguished presmolt from postsmolt pools, reflecting developmental stage differences (Figure 9).

**FIGURE 10:**
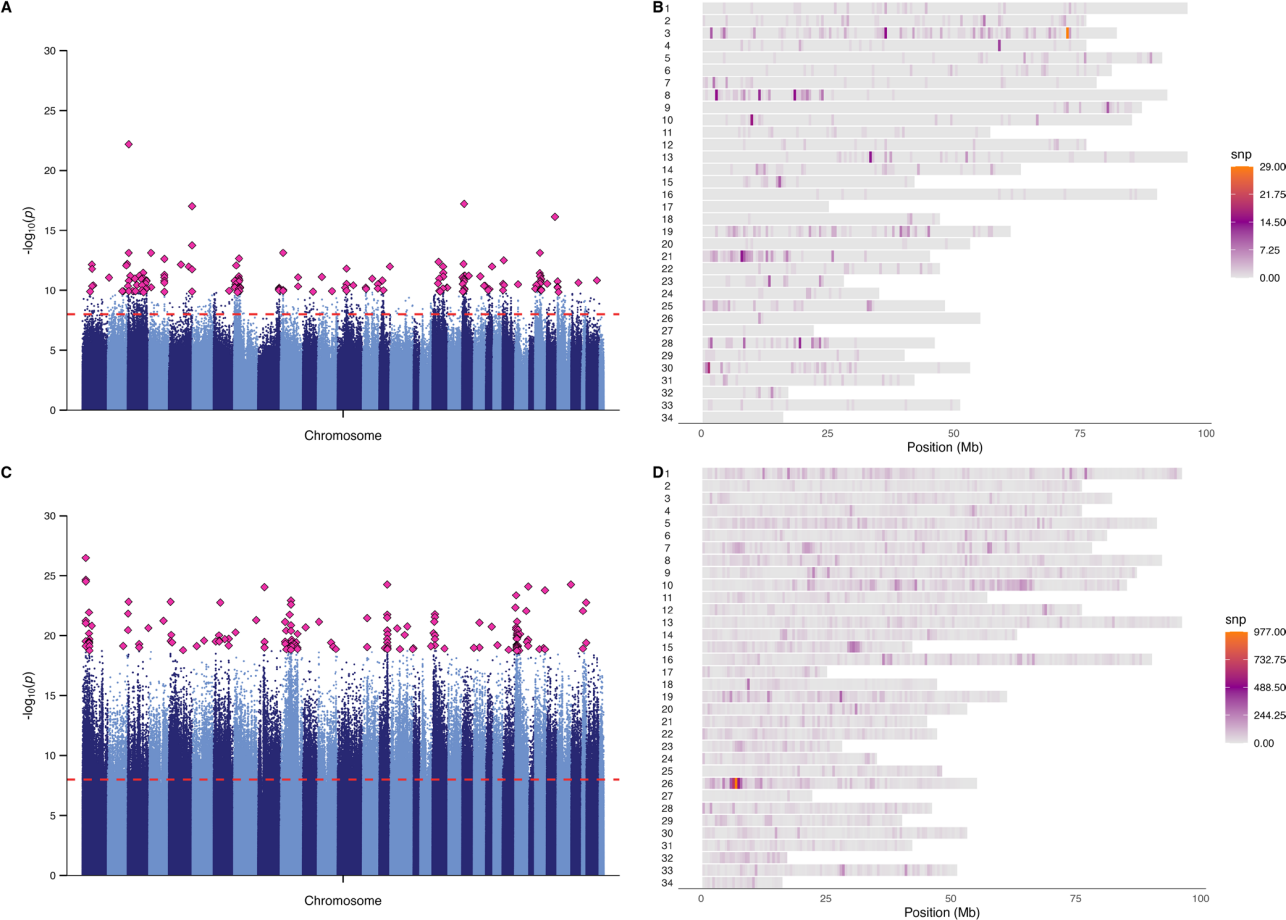
Genomic distribution of SNPs associated with thermal tolerance in the Shovelnose Chinook salmon population in freshwater and saltwater. (A, C) Manhattan plots showing –log_10_ transformed FDR‐corrected *p* values from Poolseq association analyses of top and bottom thermal tolerance performers in freshwater (A) and saltwater (C) environments. Each point represents a single SNP, with alternating chromosome colours for clarity. The horizontal dashed line marks the genome‐wide significance threshold (FDR < 1 × 10^−8^). Top 150 SNPs are highlighted as outlined maroon diamonds. (B, D) SNP density heatmaps showing the genomic distribution of significant SNPs (FDR < 1 × 10^−8^) across chromosomes in 500 kb windows for freshwater (B) and saltwater (D). Warmer colours indicate higher densities of significant SNPs, highlighting clustered genomic regions potentially involved in performance‐related thermal tolerance.

We identified 1301 SNPs significantly associated with differences in CT_max_ between top and bottom performances in freshwater and 96,557 SNPs in saltwater (FDR < 1e^−8^) (Figure 10). Strikingly, there was minimal overlap between these sets, with only *199* SNPs shared between environments, indicating that the genomic regions underlying thermal tolerance are largely specific to both environment and developmental stage. This pattern suggests that the physiological and environmental transitions associated with smoltification substantially reshape the genetic basis of thermal performance. Despite the divergence in specific loci, functional annotation revealed convergence on higher‐order biological functions. In both environments, we identified genes implicated in cellular protection, membrane organisation and immune or stress signalling.

To further compare the genomic basis of thermal tolerance between pre‐ and postsmolt stages, we examined the chromosomal distribution of significant SNPs in each environment. The SNPs associated with CT_max_ in freshwater and saltwater showed largely distinct genomic distributions, reinforcing the idea that smoltification reconfigures the genetic architecture of thermal performance. Notably, in the postsmolt data set, we identified a prominent cluster of significant SNPs on chromosome 26. Several genes in this cluster are functionally associated with cellular stress responses and protein homeostasis, including *E3 ubiquitin‐protein ligase HECW2‐like*, which is involved in protein quality control and degradation of misfolded proteins, and *serine/threonine kinase 17b*, which plays a role in apoptosis in response to stress. Additional genes of interest include *caveolae associated protein 2a*, linked to membrane stability and cellular signalling under mechanical or thermal stress, and *solute carrier family 39 member 10*, a zinc transporter implicated in oxidative stress response (Table S11).

## 4 Discussion

### 4.1 General Summary

This study demonstrates that thermal tolerance in Chinook salmon ( *Oncorhynchus tshawytscha* ) varies across populations and life‐history strategies, influenced by the physiological demands of smoltification and the transition between freshwater and marine environments. We found that both population‐specific genetic factors and developmental stage play key roles in shaping thermal resilience, revealing the complex interactions between intrinsic and extrinsic factors that influence thermal performance. Thermal tolerance is a critical determinant of survival, growth, and distribution in ectotherms, and in salmonids, this trait is shaped by the contrasting temperature and salinity conditions encountered during different life stages. By examining how life‐history variation in the timing of smoltification and the physiological transition from freshwater to saltwater influence thermal tolerance in Chinook salmon from southern British Columbia, we identified population‐specific genetic variants linked to thermal performance, reflecting the interaction between developmental processes and environmental conditions. These results highlight a polygenic genetic architecture that varies with smoltification and environmental conditions, where intrinsic genetic factors interact with environmental conditions to influence thermal resilience during this critical life‐history transition.

### 4.2 Effect of Life‐History Type and Developmental Stage on CT_max_

Population‐level variation in thermal tolerance is well documented in Chinook salmon and is widely attributed to local adaptation to environmental conditions, particularly thermal regimes experienced during critical life stages such as juvenile rearing or adult migration (Van Wert et al. 2023; Zillig, Fitzgerald, et al. 2023; Zillig, Lusardi, et al. 2023). For example, populations from warmer or more thermally variable habitats tend to exhibit higher CT_max_ and greater aerobic performance under elevated temperatures, suggesting fine‐scale adaptation to local thermal pressures. Here, we evaluated thermal tolerance across four Chinook salmon populations representing stream‐ and ocean‐type ecotypes to determine whether life‐history type and smoltification influence upper thermal limits. In general, stream‐type populations are distributed in headwater or upstream tributaries of major river systems, whereas ocean‐type populations tend to occur in lower mainstem habitats, which can differ in both average temperature and seasonal variability. Stream‐type populations remain in freshwater during the summer months when average stream temperatures are higher than sea surface temperatures and they experience multiple days during peak summer when stream temperatures exceed 19°C, indicating greater exposure to potentially stressful thermal conditions (Figure S6). In contrast, ocean‐type juveniles typically outmigrate before the annual mean temperature of their stream exceeds local sea surface temperatures. Thus, we predicted that stream‐type populations may be selected for higher thermal tolerance. However, contrary to our prediction, we found that stream‐type populations exhibited lower CT_max_ than ocean‐type populations in both freshwater and saltwater. Although hatchery conditions may influence aspects of the selective environment, all populations in this study come from integrated hatchery programs designed to reduce hatchery effects while maintaining natural selective pressures. Consequently, natural variation in freshwater thermal exposure is likely still an important factor shaping differences between stream‐ and ocean‐type juveniles.

In addition, developmental rearing conditions may also influence thermal tolerance. Experimental work in Chinook salmon has shown that early thermal environments significantly affect CT_max_ at later stages, with warm or variable rearing temperatures producing higher thermal limits relative to controls (Del Rio et al. 2021). These studies underscore the importance of evaluating intraspecific thermal physiology when predicting population resilience to climate change.

While population‐level differences in thermal tolerance have been linked to local adaptation, less is known about how thermal tolerance changes as fish transition across environments during development. In salmon, smoltification is a critical physiological transformation that prepares juveniles for migration from freshwater to saltwater, yet few studies have examined how this transition affects upper thermal limits. Most existing work emphasises how temperature influences the timing or success of smoltification (Folmar and Dickhoff 1980; Marine and Cech 2004), not how smoltification itself modulates thermal resilience. A handful of studies have explored thermal preference (Hinke et al. 2005; Sauter et al. 2001) or marine distributional limits (Walker et al. 2007; Walker and Myers 2009), but very few studies have directly assessed critical thermal limits in Chinook salmon smolts (Baker et al. 1995). Our study addresses this critical knowledge gap by directly testing the effects of smoltification on upper thermal limits across four populations. We found that smoltification consistently reduced CT_max_ in both stream‐ and ocean‐type Chinook salmon, suggesting that the physiological and osmoregulatory changes accompanying this transition reduce the capacity to withstand thermal extremes. The stream‐type Ashlu population showed the smallest decline, whereas ocean‐type populations exhibited the largest decreases, indicating that ocean‐type populations, which enter saltwater at a younger age and smaller size, may experience a greater compromise in thermal performance following smoltification. CT_max_ values measured in freshwater were not predictive of tolerance in saltwater, and greater variability was observed in saltwater across both ecotypes. This consistent salinity effect indicates that environmental context plays a central role in modulating thermal resilience, independent of life‐history strategy.

In addition to shifts in mean CT_max_, we also observed differences in the distribution of interindividual variation in thermal tolerance values across environments and developmental stages. Notably, interindividual variation in CT_max_ was consistently lower in saltwater than in freshwater, and this variability was largely concentrated at the lower end of the distribution. The upper limit of CT_max_ remained relatively consistent across populations and salinities, whereas the lower end expanded under saltwater conditions, particularly postsmoltification. This pattern aligns with the ‘plastic floors and concrete ceilings’ hypothesis (Sandblom et al. 2016), which posits that upper thermal limits are constrained by hard physiological boundaries, while lower thermal limits reflect more plastic responses to environmental and individual‐level variation. In our data, this asymmetry suggests that while some individuals are able to compensate or recover thermal performance after environmental or developmental transitions, others are more susceptible to performance loss, but few, if any, exceed a strict upper ceiling. This may reflect evolutionary constraints on protein stability, membrane integrity or cardiovascular performance at high temperatures, while allowing greater flexibility at the lower bounds depending on individual condition or plasticity. These findings imply that climate‐driven selection on upper thermal limits may be slow or constrained, whereas selection on lower performance thresholds may offer more potential for adaptive shifts or physiological buffering. Taken together, these results emphasise the importance of evaluating temperature tolerance in both freshwater and marine environments, particularly at transitional life stages like smoltification, and demonstrate that smoltification itself imposes a constraint on thermal tolerance.

### 4.3 Genomic Regions Associated With Thermal Resilience

The physiological effects of smoltification on CT_max_ suggest that life history plays a key role in shaping thermal resilience, but the extent to which this variation reflects differentiation or other regulatory mechanisms remains unclear. To address this, we asked whether we could identify genomic regions associated with increased thermal resilience and whether these associations are consistent across populations with contrasting migratory strategies.

Our analyses revealed substantial population‐specific patterns: Stream‐type populations (Ashlu and Shovelnose) exhibited stronger associations between genotype and thermal tolerance than ocean‐type populations (Chehalis and Chilliwack), consistent with the idea that prolonged freshwater residence may impose stronger selection for thermal resilience. GO enrichment analyses revealed that each population had a distinct set of biological processes associated with thermal performance. Both ocean‐type populations (Chilliwack and Chehalis) showed enrichment for signalling and transport pathways, including cytokine production, the calcineurin‐NFAT signalling cascade and retrograde vesicle transport. The NFAT signalling pathway, which regulates Ca^2+^‐dependent transcription and osmo‐sensing, has been implicated in gill remodelling and salinity‐induced transcriptional control during smoltification (Iversen et al. 2021; Lorgen et al. 2017). The potential for both salinity and temperature changes to challenge cellular homeostasis through Ca^2+^‐mediated signalling suggests that NFAT pathways may provide a shared Ca^2+^‐dependent regulatory link between osmoregulatory and thermal stress responses during smoltification. The retrograde response, a mitochondria‐to‐nucleus signalling pathway, plays a broader role in maintaining cellular homeostasis and stress resilience through modulation of metabolic and quality‐control networks (Jazwinski 2014). In contrast, stream‐type populations (Ashlu and Shovelnose) were enriched for a broader diversity of processes related to transcription, translation and cellular stress responses, such as apoptotic signalling in response to ER stress, synaptic function, oxidative stress response and the unfolded protein response. These processes work in concert to protect cells from thermal stress, ensuring protein stability and mitochondrial function (Ern et al. 2023; Esmaeili et al. 2025). While stress‐related pathways were present across both life‐history types, stream‐type populations exhibited additional enrichment for neuronal signalling and structural organisation processes. These results support the interpretation that thermal tolerance is a complex, polygenic trait, shaped by both life history and population‐specific selective pressures, with different populations relying on distinct physiological mechanisms to cope with thermal stress.

The population‐specific nature of these associations is consistent with findings from other fish species, where thermal tolerance has been shown to involve polygenic architectures. For example, genome‐wide association studies in olive and Japanese flounder have identified dozens of loci linked to survival under thermal stress, with nearby genes involved in diverse biological processes including metabolic, neural, immune and cellular regulatory pathways (San et al. 2024; Udayantha et al. 2023). Similarly, GWAS in northern pike revealed candidate genes linked to ion regulation and general stress response (Jiang et al. 2022). In Atlantic salmon, thermal tolerance has been shown to be moderately to highly heritable (Benfey et al. 2024; Gonen et al. 2024), yet consistent major‐effect loci have not been identified, underscoring the polygenic nature of thermal tolerance in fishes. In line with these findings, our study demonstrates limited overlap in gene‐level associations across Chinook salmon populations but broad convergence in higher‐order biological processes related to stress response, cellular signalling and proteostasis, with a particular emphasis on cytokine production, ER stress signalling and mitochondrial function.

Together, these data indicate that thermal tolerance in Chinook salmon is a polygenic trait and this genetic basis is largely population‐specific. However, the same physiological systems, including stress response, signalling and proteostasis, are consistently involved, while the underlying genetic architecture varies with life history and local environmental pressures. These findings highlight the importance of developing population‐tailored genomic tools to support conservation and climate resilience efforts in salmon and other ecologically or economically important species.

### 4.4 Association Between Gill Gene Expression and Thermal Resilience

Identifying molecular indicators of thermal tolerance is likely to aid in predicting how ectothermic species will respond to rising temperatures. We therefore asked whether baseline gene expression in gill tissue could predict CT_max_ among presmolt juveniles. Salmonids, in particular, are highly sensitive to temperature fluctuations (Richter and Kolmes 2005). Much of our current understanding of the mechanisms underlying thermal resilience in Chinook salmon comes from studies examining acute responses to heat stress, including shifts in metabolic and oxidative pathways (Clark et al. 2008; Dimos et al. 2023; Farrell 2009) and the activation of molecular pathways and gene expression responses (Houde et al. 2019; Marcoli et al. 2023; Tomalty et al. 2015; von Biela et al. 2020). These studies provide important insight into short‐term stress responses, but thermal tolerance ultimately reflects a complex, integrative network of molecular, physiological and ecological adaptations that span multiple levels of biological organisation, from gene regulation to whole‐organism performance and population‐level processes (Metzger et al. 2024; Thorogood et al. 2023). Resilience also encompasses the ability to maintain performance under environmental change (Metzger et al. 2024), which may involve subtle or constitutive gene expression differences overlooked in short‐term stress studies. Our study used a performance‐based approach to identify resilient individuals based on their physiological capacity to tolerate high temperatures.

By integrating transcriptomics with genomics, we sought to identify molecular mechanisms underlying individual thermal performance. Population‐level differences in transcript abundance were the dominant source of variation, and the ability of baseline gene expression to predict thermal tolerance varied by population. Stream‐type populations (Ashlu and Shovelnose) showed clearer differentiation between top and bottom thermal performers compared to ocean‐type populations (Chehalis and Chilliwack), suggesting that gene expression was more strongly linked to thermal performance in stream‐type populations, where we observed distinct expression patterns and a higher number of differentially expressed genes (DEGs).

Among the top genes associated with thermal performance, STARD7, ZNF711 and TNFRSF3 emerge as promising candidates for predicting thermal performance. Although their roles in thermal tolerance in fishes are not well characterised, these genes are involved in cellular stress responses in other systems and may represent candidates for future study (Flores‐Martín et al. 2016; Zhao et al. 2022; X. Liu et al. 2014; Kooy 2022). Shovelnose exhibited significant GO enrichment for protein ubiquitination and mRNA splicing. These processes are essential for maintaining proteostasis and regulating gene expression, and both play central roles in the temperature stress response of salmonids (Healy and Schulte 2019; X.‐X. Liu et al. 2022; Maxwell et al. 2021; Sheng et al. 2024; Thorstensen et al. 2022).

In contrast, Ashlu, Chehalis and Chilliwack showed no enriched GO terms, likely due to smaller DEG sets or greater variability in gene expression among individuals. To explore potential biological relevance in these populations, we examined individual candidate genes with consistent expression differences between performance groups. In Ashlu, candidate genes included LRSAM1, an E3 ubiquitin ligase involved in cellular proteostasis (Mishra et al. 2021); IRE1α, a central sensor in the unfolded protein response that maintains ER protein folding homeostasis (Belyy et al. 2022); DMGDH, a gene linked to mitochondrial redox metabolism and protection against oxidative stress from elevated temperatures (Cheruiyot et al. 2021); and BbX, a High‐Mobility Group (HMG) protein previously implicated in the temperature stress response in fish (Martínez et al. 2023). In Chehalis, CIAO1, a chaperone supporting iron–sulfur cluster assembly, was differentially expressed (Martins et al. 2020). In Chilliwack, high performers upregulated a ZNF574‐like transcription factor, part of the ribosome assembly surveillance pathway (Akers et al. 2025), while FSTL1B, a glycoprotein modulating stress and inflammation via AMPK signalling, was downregulated (H. Liu et al. 2023).

Despite differences in analytical approaches and DEG counts, common functional themes emerged across populations, particularly in genes involved in protein regulation, oxidative stress response and transcriptional control. Notably, several identified genes are transcription factors or upstream regulators, suggesting that population‐specific variation in a small number of regulatory nodes could produce broad downstream effects. This pattern is consistent with the complex, polygenic architecture of thermal performance, where diverse combinations of regulatory and stress–response pathways may contribute to individual and population‐level resilience.

Together, these findings suggest that baseline gene expression in gill tissue provides limited but informative insight into individual variation in thermal tolerance. While several genes showed consistent expression differences between high‐ and low‐performing juveniles, these relationships were not uniformly strong or shared across populations. This pattern indicates that constitutive gene expression may contribute to thermal resilience in some contexts, but its predictive value is likely population‐specific and influenced by other genetic and environmental factors. Overall, our results highlight potential molecular candidates and pathways for future investigation rather than definitive transcriptional predictors of thermal performance.

### 4.5 Genomic Signatures of Thermal Tolerance Across Life Stages

We next asked whether the molecular predictors of thermal tolerance shift as juveniles transition from freshwater to marine environments. Our results reveal that the genomic regions associated with thermal tolerance differ markedly between freshwater and saltwater environments in Shovelnose Chinook salmon, indicating that the molecular architecture underlying thermal performance shifts following smoltification. This suggests that the physiological and environmental transition from freshwater to marine habitats plays a pivotal role in shaping the genetic basis of thermal resilience. Smoltification is a complex developmental process involving extensive remodelling of ion regulation, metabolism and endocrine pathways to prepare juveniles for saltwater entry. These changes, combined with the distinct thermal properties and osmotic challenges of saltwater, likely impose new selective pressures on thermal tolerance mechanisms.

Despite minimal overlap in the specific genomic regions identified as associated with thermal tolerance in each environment, functional analyses point to convergence in higher‐level biological processes. Several candidate genes identified across both conditions implicate a coordinated response to thermal stress involving cellular protection, structural integrity and immune signalling. For example, striated muscle preferentially expressed protein kinase‐like and caveolae associated protein 2a may support muscle function and membrane organisation under heat stress (Luo et al. 2021; Parton et al. 2020), while C‐X‐C chemokine receptor type 1 and tomoregulin‐2 reflect neuroendocrine and androgen‐regulated components of stress‐responsive signalling (Overcash et al. 2013; Pijanowski et al. 2019). Additionally, E3 ubiquitin‐protein ligase HECW2‐like and serine/threonine kinase 17b, involved in proteostasis and apoptotic regulation, suggest a role in maintaining cellular homeostasis under stress (Rotin and Kumar 2009; Sanjo et al. 1998).

These data suggest that the genetic associations with thermal tolerance vary across life stages and salinity environments. Although the specific loci associated with thermal performance differ between freshwater and saltwater, convergence in functional pathways indicates that similar physiological processes are recruited to maintain resilience under changing conditions. Taken together, these results underscore the importance of considering developmental and environmental context when evaluating the genetic basis of thermal tolerance.

## 5 Conclusion

Our integrative analysis of Chinook salmon thermal tolerance across developmental stages, environments and populations reveals that thermal resilience is shaped by a complex interplay among life‐history strategy, physiological changes during smoltification and population‐specific molecular signatures. The transition to saltwater appears to amplify interindividual variation in thermal tolerance, revealing physiological differences that are less apparent in freshwater. These findings suggest that environmental context, particularly the shift from freshwater to saltwater, plays a critical role in shaping thermal performance. Moreover, our identification of population‐specific genetic regions and gene expression patterns associated with thermal resilience underscores the importance of both intrinsic biological processes and local adaptation in shaping species responses to climate change. Together, these findings suggest that thermal resilience may be attributed to similar physiological frameworks implemented through diverse, population‐specific molecular mechanisms. The polygenic nature of thermal tolerance indicates that developing predictive bioassays will be challenging, though targeted approaches, such as SNP panels capturing loci with the largest effects combined with physiological measurements, could help identify thermally resilient individuals. This work provides a valuable foundation for refining conservation and management strategies under warming scenarios and demonstrates the utility of integrative, performance‐based genomic approaches for identifying thermally resilient individuals and populations in salmon and other ecologically sensitive species.

## Author Contributions

D.C.H.M. conducted the experiments, performed the laboratory work and carried out the bioinformatic analyses. D.C.H.M. also contributed to project planning and experimental design beyond the scope of the original grant and wrote the first draft of the manuscript. T.M.H. conceived the study, assisted with experimental trials, provided input on data analysis and provided manuscript revisions. K.W. contributed to study design, was responsible for animal husbandry and assisted with experimental trials and manuscript revisions. P.M.S. contributed to project design and supervision and provided feedback on the manuscript.

## Funding

This work was funded by Genome BC through the GIRAFF program (GIR008). Sequencing support was provided by Génome Québec. K.W. was supported by the Canadian Regulatory System for Biotechnology through Fisheries and Oceans Canada. P.M.S. is supported by a Canada Research Chair (CRC‐2021‐00040).

## Conflicts of Interest

The authors declare no conflicts of interest.

## Supporting information


**Data S1:** mec70233‐sup‐0001‐Supinfo1.txt.

**Table S1:** CT_max_ summary statistics for four population of Chinook Salmon pre‐ and postsmoltification.
**Table S2:** Fixed effects from linear mixed model predicting CTmax.
**Table S3:** Post hoc pairwise comparisons of CTmax (Tukey‐adjusted).
**Table S4:** Weight, fork length, and condition factor of Chinook salmon by population.
**Table S5:** Poolseq GO enrichment results—Ashlu.
**Table S6:** Poolseq GO enrichment results—Shovelnose.
**Table S7:** Poolseq GO enrichment results—Chehalis.
**Table S8:** Poolseq GO enrichment analysis—Chilliwack.
**Table S9:** Overlapping interquartile range for DEGs between top and bottom performers.
**Table S10:** GO enrichment results for Shovelnose DEGs.
**Table S11:** Annotated Genes within SNP cluster on Linkage Group 26.
**Figure S1:** Thermal tolerance (CTmax) variation across populations in freshwater and saltwater conditions, grouped by sex. Boxplots show the distribution of CTmax values for male (blue) and female (red) individuals across four Chinook salmon populations: Ashlu, Shovelnose, Chehalis and Chilliwack. The *y*‐axis represents the CTmax temperature (°C). The plot is split by salinity along the *x*‐axis (freshwater and saltwater). Each box represents the interquartile range (IQR), with the median marked by a horizontal line. Whiskers extend to the 1.5 × IQR, and outliers are shown as individual points.
**Figure S2:** Relationship between CTMax in freshwater (FW) and seawater (SW).
**Figure S3:** Principal component analysis of Chinook salmon allele frequencies along secondary dimensions (PC2–PC4) associated with thermal tolerance. (A) PC2 and PC3 separate individuals by population, with no clear clustering by thermal tolerance, indicating that population‐level structure remains a dominant signal in PC3. (B) PC3 captures population structure while PC4 distinguishes top‐ and bottom‐performing individuals from the Shovelnose population, one of the two populations with a stream‐type life history, representing the first axis that begins to capture variation associated with thermal performance. Each point represents a pooled sequencing sample comprising individuals from the same population and performance group, with colours indicating performance (Top vs. Bottom) and shapes denoting population of origin. Populations are distinguished by colour: Ashlu (slate blue), Chehalis (green), Chilliwack (orange), and Shovelnose (magenta). Performance groups are distinguished by shape: Filled symbols indicate top performers and open symbols indicate bottom performers.
**Figure S4:** Manhattan plots of –log10(s) from Fisher's exact tests comparing allele frequencies between high and low thermal performers in four Chinook salmon populations.
Each panel shows genome‐wide results from poolseq‐based differentiation analyses between the top and bottom thermal tolerance groups for a given population: (A) Ashlu, (B) Chehalis, (C) Shovelnose, and (D) Chilliwack. SNPs are plotted by genomic position across concatenated chromosomes, with alternating colours distinguishing chromosomes. The horizontal dotted line indicates the genome‐wide significance threshold (−log_10_(*p*) = 1e^−8^). Peaks above this threshold represent SNPs significantly differentiated between thermal performance groups, potentially reflecting loci associated with thermal tolerance.
**Figure S5:** Overlap of significantly differentiated SNPs between top and bottom thermal performers across four Chinook salmon populations. Venn diagram showing the number of unique and shared SNPs identified by Fisher's exact tests in poolseq data from the Ashlu, Chehalis, Shovelnose, and Chilliwack populations. SNPs were considered significantly differentiated if they exceeded the population‐specific –log10(*p*) significance threshold in the Manhattan plots (see Figure S4). Most loci are population‐specific, with very limited overlap among populations, suggesting distinct genomic responses associated with thermal tolerance. Shovelnose exhibited the largest number of significant loci, while only four SNPs were shared between Chilliwack and Ashlu and a single SNP was shared between Shovelnose and Chilliwack. No loci were shared across more than two populations.
**Figure S6:** Daily mean water temperatures for the Harrison River (red) and Squamish River (light blue) from 1971 to 2000. Data were obtained from the Salmon Climate Impacts Portal (Pacific Climate Impacts Consortium; https://services.pacificclimate.org/scip/app/). Mean monthly sea surface temperatures are shown in dark blue, derived from measurements at Race Rocks Lighthouse (BC Lightstations). The shaded grey region indicates the typical freshwater outmigration period for Chinook salmon (see Wilson and Peacock 2025, for details). Inset: number of days in 2025 that river temperatures exceeded 19°C, based on Salmon Climate Impacts Portal data. This figure illustrates historical river thermal regimes relative to sea surface temperatures and highlights recent high‐temperature events in freshwater habitats.

## Data Availability

Raw sequencing data have been deposited in the NCBI Sequence Read Archive (SRA) under accession number PRJNA1274953, https://www.ncbi.nlm.nih.gov/sra/. Processed data files and supporting metadata are available as Supporting Information. Code used for bioinformatic processing and analysis is available on GitHub at https://github.com/D‐Metzger/GIRAFF_poolseq.
