# Supplementary material for: Life History and Smoltification Shape Genomic Signatures of Thermal Tolerance in Chinook Salmon
Source: Mol Ecol. 2026 Jan 12;35(1):e70233. doi: 10.1111/mec.70233 (PMC12794147; doi:10.1111/mec.70233)
Supplement: Supplementary file 2 — Table S1: CTmax summary statistics for four population of Chinook Salmon pre‐ and postsmoltification. Table S2: Fixed effects from linear mixed model predicting CTmax. Table S3: Post hoc pairwise comparisons of CTmax (Tukey‐adjusted). Table S4: Weight, fork length, and condition factor of Chinook salmon by population. Table S5: Poolseq GO enrichment results—Ashlu. Table S6: Poolseq GO enrichment results—Shovelnose. Table S7: Poolseq GO enrichment results—Chehalis. Table S8: Poolseq GO enrichment analysis—Chilliwack. Table S9: Overlapping interquartile range for DEGs between top and bottom performers. Table S10: GO enrichment results for Shovelnose DEGs. Table S11: Annotated Genes within SNP cluster on Linkage Group 26. Figure S1: Thermal tolerance (CTmax) variation across populations in freshwater and saltwater conditions, grouped by sex. Boxplots show the distribution of CTmax values for male (blue) and female (red) individuals across four Chinook salmon populations: Ashlu, Shovelnose, Chehalis and Chilliwack. The y‐axis represents the CTmax temperature (°C). The plot is split by salinity along the x‐axis (freshwater and saltwater). Each box represents the interquartile range (IQR), with the median marked by a horizontal line. Whiskers extend to the 1.5 × IQR, and outliers are shown as individual points. Figure S2: Relationship between CTMax in freshwater (FW) and seawater (SW). Figure S3: Principal component analysis of Chinook salmon allele frequencies along secondary dimensions (PC2–PC4) associated with thermal tolerance. (A) PC2 and PC3 separate individuals by population, with no clear clustering by thermal tolerance, indicating that population‐level structure remains a dominant signal in PC3. (B) PC3 captures population structure while PC4 distinguishes top‐ and bottom‐performing individuals from the Shovelnose population, one of the two populations with a stream‐type life history, representing the first axis that begins to capture variation associated with th [file MEC-35-e70233-s002.docx]

**Table S1:** **CT_max_ summary statistics for four population of Chinook Salmon pre- and post-smoltification.**

Table S2. Fixed effects from linear mixed model predicting CTmax

| Term | Estimate | Std. Error | df | t value | p value | 95% CI Lower | 95% CI Upper |
| --- | --- | --- | --- | --- | --- | --- | --- |
| *Intercept* | 27.281 | 0.050 | 6.4 | 541.697 | 0.000 | 27.160 | 27.402 |
| *Shovelnose (vs. Ashlu)* | -0.057 | 0.035 | 3,746.0 | -1.629 | 0.103 | -0.125 | 0.012 |
| *Chehalis (vs. Ashlu)* | 0.502 | 0.035 | 3,746.0 | 14.471 | 0.000 | 0.434 | 0.570 |
| *Chilliwack (vs. Ashlu)* | 0.540 | 0.035 | 3,746.2 | 15.467 | 0.000 | 0.472 | 0.609 |
| *Saltwater (vs. Freshwater)* | -0.349 | 0.036 | 3,746.0 | -9.747 | 0.000 | -0.419 | -0.278 |
| *Shovelnose × Saltwater* | -0.174 | 0.050 | 3,746.1 | -3.483 | 0.001 | -0.272 | -0.076 |
| *Chehalis × Saltwater* | -0.193 | 0.050 | 3,746.0 | -3.896 | 0.000 | -0.291 | -0.096 |
| *Chilliwack × Saltwater* | -0.220 | 0.051 | 3,746.2 | -4.308 | 0.000 | -0.320 | -0.120 |

Table S3. Post hoc pairwise comparisons of CTmax (Tukey-adjusted)

| Contrast | Estimate | SE | z | p |
| --- | --- | --- | --- | --- |
| Ashlu FW - Shovelnose FW | 0.057 | 0.035 | 1.629 | 0.7326 |
| Ashlu FW - Chehalis FW | -0.502 | 0.035 | -14.471 | <0.0001 |
| Ashlu FW - Chilliwack FW | -0.540 | 0.035 | -15.467 | <0.0001 |
| Ashlu FW - Ashlu SW | 0.348 | 0.035 | 9.747 | <0.0001 |
| Ashlu FW - Shovelnose SW | 0.579 | 0.035 | 16.565 | <0.0001 |
| Ashlu FW - Chehalis SW | 0.040 | 0.035 | 1.144 | 0.9471 |
| Ashlu FW - Chilliwack SW | 0.028 | 0.035 | 0.770 | 0.9946 |
| Shovelnose FW - Chehalis FW | -0.559 | 0.036 | -16.192 | <0.0001 |
| Shovelnose FW - Chilliwack FW | -0.597 | 0.036 | -17.184 | <0.0001 |
| Shovelnose FW - Ashlu SW | 0.292 | 0.036 | 8.201 | <0.0001 |
| Shovelnose FW - Shovelnose SW | 0.522 | 0.036 | 15.018 | <0.0001 |
| Shovelnose FW - Chehalis SW | -0.017 | 0.036 | -0.491 | 0.9997 |
| Shovelnose FW - Chilliwack SW | -0.029 | 0.036 | -0.790 | 0.9937 |
| Chehalis FW - Chilliwack FW | -0.038 | 0.036 | -1.098 | 0.9575 |
| Chehalis FW - Ashlu SW | 0.851 | 0.037 | 23.992 | <0.0001 |
| Chehalis FW - Shovelnose SW | 1.081 | 0.035 | 31.200 | <0.0001 |
| Chehalis FW - Chehalis SW | 0.542 | 0.035 | 15.731 | <0.0001 |
| Chehalis FW - Chilliwack SW | 0.530 | 0.035 | 14.660 | <0.0001 |
| Chilliwack FW - Ashlu SW | 0.889 | 0.035 | 24.914 | <0.0001 |
| Chilliwack FW - Shovelnose SW | 1.119 | 0.035 | 32.085 | <0.0001 |
| Chilliwack FW - Chehalis SW | 0.580 | 0.035 | 16.729 | <0.0001 |
| Chilliwack FW - Chilliwack SW | 0.568 | 0.035 | 15.619 | <0.0001 |
| Ashlu SW - Shovelnose SW | 0.231 | 0.036 | 6.455 | <0.0001 |
| Ashlu SW - Chehalis SW | -0.309 | 0.036 | -8.695 | <0.0001 |
| Ashlu SW - Chilliwack SW | -0.321 | 0.036 | -8.618 | <0.0001 |
| Shovelnose SW - Chehalis SW | -0.539 | 0.036 | -15.536 | <0.0001 |
| Shovelnose SW - Chilliwack SW | -0.551 | 0.036 | -15.131 | <0.0001 |
| Chehalis SW - Chilliwack SW | -0.012 | 0.036 | -0.322 | 1 |

**Table S4: Weight, fork length, and condition factor of Chinook salmon by population**

| **Population** | **Weight (g)** | **Fork Length (cm)** | **Condition Factor** |
| --- | --- | --- | --- |
| Shovelnose | 21.4 ± 5.34 | 12.2 ± 0.97 | 1.171 ± 0.089 |
| Ashlu | 17.8 ± 3.69 | 11.7 ± 0.69 | 1.082 ± 0.06 |
| Chilliwack | 10.8 ± 1.77 | 9.8 ± 0.48 | 1.127 ± 0.063 |
| Chehalis | 8 ± 1.66 | 8.8 ± 0.6 | 1.15 ± 0.081 |

**Table S5: Poolseq GO enrichment results - Ashlu**

| Category | p-value | Number in Category | Total in Category | Term |
| --- | --- | --- | --- | --- |
| GO:0070059 | 0.0002371931 | 3 | 14 | intrinsic apoptotic signaling pathway in response to endoplasmic reticulum stress |
| GO:0048704 | 0.0008206413 | 3 | 21 | embryonic skeletal system morphogenesis |
| GO:0009952 | 0.0013934824 | 5 | 93 | anterior/posterior pattern specification |
| GO:0007187 | 0.0021006365 | 3 | 29 | G protein-coupled receptor signaling pathway, coupled to cyclic nucleotide second messenger |
| GO:0048790 | 0.0066295588 | 2 | 13 | maintenance of presynaptic active zone structure |
| GO:0009134 | 0.0087269040 | 2 | 15 | nucleoside diphosphate catabolic process |
| GO:0071880 | 0.0099829693 | 4 | 95 | adenylate cyclase-activating adrenergic receptor signaling pathway |
| GO:0000165 | 0.0102141597 | 2 | 17 | MAPK cascade |
| GO:0005979 | 0.0123116745 | 2 | 18 | regulation of glycogen biosynthetic process |
| GO:0018105 | 0.0154845768 | 5 | 162 | peptidyl-serine phosphorylation |
| GO:0000463 | 0.0160226054 | 2 | 20 | maturation of LSU-rRNA from tricistronic rRNA transcript (SSU-rRNA, 5.8S rRNA, LSU-rRNA) |
| GO:0015749 | 0.0226207462 | 2 | 26 | monosaccharide transmembrane transport |
| GO:0048167 | 0.0248840685 | 2 | 27 | regulation of synaptic plasticity |
| GO:0050852 | 0.0266525962 | 2 | 29 | T cell receptor signaling pathway |
| GO:0000027 | 0.0269521708 | 2 | 29 | ribosomal large subunit assembly |
| GO:0007274 | 0.0295669161 | 2 | 29 | neuromuscular synaptic transmission |
| GO:0006376 | 0.0310563463 | 2 | 31 | mRNA splice site recognition |
| GO:0006397 | 0.0341993105 | 3 | 78 | mRNA processing |
| GO:0015908 | 0.0448472023 | 2 | 38 | fatty acid transport |
| GO:0006816 | 0.0485427925 | 2 | 39 | calcium ion transport |
| GO:0006891 | 0.0498067549 | 2 | 40 | intra-Golgi vesicle-mediated transport |

**Table S6. Poolseq GO enrichment results – Shovelnose**

| Category | p-value | Number in Category | Total in Category | Term |
| --- | --- | --- | --- | --- |
| GO:0048870 | 0.002287291 | 8 | 30 | cell motility |
| GO:0002084 | 0.002403333 | 6 | 18 | protein depalmitoylation |
| GO:1902476 | 0.002404545 | 13 | 66 | chloride transmembrane transport |
| GO:0009952 | 0.003446909 | 16 | 93 | anterior/posterior pattern specification |
| GO:0045596 | 0.003991679 | 4 | 9 | negative regulation of cell differentiation |
| GO:0001649 | 0.005420948 | 5 | 15 | osteoblast differentiation |
| GO:0055064 | 0.005528239 | 6 | 21 | chloride ion homeostasis |
| GO:0055075 | 0.005528239 | 6 | 21 | potassium ion homeostasis |
| GO:0010923 | 0.006239548 | 4 | 10 | negative regulation of phosphatase activity |
| GO:0046513 | 0.007061776 | 9 | 43 | ceramide biosynthetic process |
| GO:0006813 | 0.008814543 | 11 | 60 | potassium ion transport |
| GO:0030705 | 0.008933687 | 6 | 23 | cytoskeleton-dependent intracellular transport |
| GO:0046475 | 0.009798402 | 5 | 17 | glycerophospholipid catabolic process |
| GO:0006884 | 0.011177037 | 6 | 24 | cell volume homeostasis |
| GO:0031124 | 0.013034756 | 4 | 12 | mRNA 3'-end processing |
| GO:0006414 | 0.013827371 | 6 | 25 | translational elongation |
| GO:0006368 | 0.014520640 | 8 | 40 | transcription elongation by RNA polymerase II |
| GO:0002224 | 0.015697581 | 7 | 33 | toll-like receptor signaling pathway |
| GO:0045116 | 0.016332253 | 5 | 19 | protein neddylation |
| GO:0035335 | 0.017739513 | 4 | 13 | peptidyl-tyrosine dephosphorylation |
| GO:0006096 | 0.018763563 | 9 | 50 | glycolytic process |
| GO:0030514 | 0.019057133 | 8 | 42 | negative regulation of BMP signaling pathway |
| GO:0007156 | 0.019759943 | 35 | 298 | homophilic cell adhesion via plasma membrane adhesion molecules |
| GO:0006979 | 0.027236475 | 9 | 53 | response to oxidative stress |
| GO:0016064 | 0.029603020 | 4 | 15 | immunoglobulin mediated immune response |
| GO:0015804 | 0.029733186 | 4 | 15 | neutral amino acid transport |
| GO:0051896 | 0.029825765 | 4 | 15 | regulation of phosphatidylinositol 3-kinase/protein kinase B signal transduction |
| GO:0033554 | 0.029936649 | 5 | 22 | cellular response to stress |
| GO:0001731 | 0.029945343 | 4 | 15 | formation of translation preinitiation complex |
| GO:0003333 | 0.031447255 | 8 | 46 | amino acid transmembrane transport |
| GO:0006606 | 0.034788789 | 11 | 73 | protein import into nucleus |
| GO:0016567 | 0.035060615 | 28 | 239 | protein ubiquitination |
| GO:0008285 | 0.037005401 | 7 | 39 | negative regulation of cell population proliferation |
| GO:0000226 | 0.038314848 | 22 | 180 | microtubule cytoskeleton organization |
| GO:0030968 | 0.039819633 | 8 | 48 | endoplasmic reticulum unfolded protein response |
| GO:0007188 | 0.041557584 | 11 | 75 | adenylate cyclase-modulating G protein-coupled receptor signaling pathway |
| GO:0000132 | 0.041599727 | 5 | 24 | establishment of mitotic spindle orientation |
| GO:0009953 | 0.041900138 | 5 | 24 | dorsal/ventral pattern formation |
| GO:0045454 | 0.041937074 | 5 | 24 | cell redox homeostasis |
| GO:0006351 | 0.043428030 | 12 | 85 | DNA-templated transcription |
| GO:0019941 | 0.044787495 | 4 | 17 | modification-dependent protein catabolic process |
| GO:0000289 | 0.049010544 | 5 | 25 | nuclear-transcribed mRNA poly(A) tail shortening |
| GO:0051603 | 0.049374634 | 9 | 59 | proteolysis involved in protein catabolic process |

**Table S7. Poolseq GO enrichment results – Chehalis**

| Category | p-value | Number in Category | Total in Category | Term |
| --- | --- | --- | --- | --- |
| GO:0015910 | 0.00003001672 | 2 | 9 | long-chain fatty acid import into peroxisome |
| GO:0042760 | 0.00004607631 | 2 | 11 | very long-chain fatty acid catabolic process |
| GO:0006635 | 0.00037688141 | 2 | 31 | fatty acid beta-oxidation |
| GO:2001234 | 0.01183112757 | 1 | 13 | negative regulation of apoptotic signaling pathway |
| GO:0007254 | 0.01456452292 | 1 | 16 | JNK cascade |
| GO:0031145 | 0.01485174092 | 1 | 17 | anaphase-promoting complex-dependent catabolic process |
| GO:0001816 | 0.02012602472 | 1 | 22 | cytokine production |
| GO:0033173 | 0.02084534027 | 1 | 23 | calcineurin-NFAT signaling cascade |
| GO:0045454 | 0.02183676160 | 1 | 24 | cell redox homeostasis |
| GO:0007162 | 0.02561889746 | 1 | 28 | negative regulation of cell adhesion |
| GO:1902287 | 0.02561889746 | 1 | 28 | semaphorin-plexin signaling pathway involved in axon guidance |
| GO:0016485 | 0.02654691789 | 1 | 29 | protein processing |
| GO:0030336 | 0.02720959806 | 1 | 30 | negative regulation of cell migration |
| GO:0006082 | 0.02831747686 | 1 | 31 | organic acid metabolic process |
| GO:0006805 | 0.02831747686 | 1 | 31 | xenobiotic metabolic process |
| GO:0006891 | 0.03601210158 | 1 | 40 | intra-Golgi vesicle-mediated transport |
| GO:0008286 | 0.03911708537 | 1 | 43 | insulin receptor signaling pathway |
| GO:0050772 | 0.03958838298 | 1 | 44 | positive regulation of axonogenesis |
| GO:0006890 | 0.04619182427 | 1 | 51 | retrograde vesicle-mediated transport, Golgi to endoplasmic reticulum |
| GO:0046777 | 0.04683177089 | 1 | 52 | protein autophosphorylation |

**Table S8. Poolseq GO enrichment analysis – Chilliwack**

| Category | p-value | Number in Category | Total in Category | Term |
| --- | --- | --- | --- | --- |
| GO:0007157 | 0.00002586831 | 3 | 74 | heterophilic cell-cell adhesion via plasma membrane cell adhesion molecules |
| GO:0048666 | 0.00021666716 | 2 | 28 | neuron development |
| GO:2001234 | 0.00990486805 | 1 | 13 | negative regulation of apoptotic signaling pathway |
| GO:0007166 | 0.01097211519 | 2 | 207 | cell surface receptor signaling pathway |
| GO:0019752 | 0.01240095697 | 1 | 16 | carboxylic acid metabolic process |
| GO:0036211 | 0.01456430083 | 1 | 19 | protein modification process |
| GO:0006357 | 0.01683882924 | 6 | 2,771 | regulation of transcription by RNA polymerase II |
| GO:0001816 | 0.01690093716 | 1 | 22 | cytokine production |
| GO:0033173 | 0.01746864953 | 1 | 23 | calcineurin-NFAT signaling cascade |
| GO:0008333 | 0.01969043308 | 1 | 26 | endosome to lysosome transport |
| GO:0016485 | 0.02232701354 | 1 | 29 | protein processing |
| GO:0006891 | 0.03025248347 | 1 | 40 | intra-Golgi vesicle-mediated transport |
| GO:0006890 | 0.03889419236 | 1 | 51 | retrograde vesicle-mediated transport, Golgi to endoplasmic reticulum |

**Table S9. Overlapping interquartile range for DEGs between top and bottom performers**

| Gene | Gene name | IQR Overlap | logFC | logCPM | F | PValue | FDR |
| --- | --- | --- | --- | --- | --- | --- | --- |
| LOC112219403 | STARD7 | 0.107 | 0.385 | 0.833 | 24.587 | 3.05e-06 | 0.022 |
| LOC112231283 | ZNF711 | 0.110 | -0.228 | 2.497 | 19.473 | 2.66e-05 | 0.086 |
| LOC112249066 | TNFRSF3 | 0.189 | -0.421 | 0.239 | 19.673 | 2.45e-05 | 0.086 |
| LOC121843856 | uncharacterized | 0.384 | -1.179 | 0.253 | 19.894 | 2.22e-05 | 0.086 |
| LOC112244799 | cacna1da | 0.681 | 1.542 | 0.934 | 24.758 | 2.85e-06 | 0.022 |
| LOC112248654 | ARHGAP25 | 0.747 | 1.926 | 6.634 | 24.974 | 2.60e-06 | 0.022 |
| LOC112262603 | uncharacterized | 0.838 | -1.784 | 0.869 | 24.018 | 3.87e-06 | 0.022 |
| LOC112265023 | uncharacterized | 0.850 | -0.939 | 2.282 | 20.377 | 1.80e-05 | 0.086 |
| f2 | F2 | 0.875 | 1.676 | 0.793 | 29.981 | 3.48e-07 | 0.010 |

**Table S10. GO enrichment results for Shovelnose DEGs**

| GO_ID | Term | Ontology | P_value | FDR | Num_DEGs | Num_genes |
| --- | --- | --- | --- | --- | --- | --- |
| GO:0006888 | endoplasmic reticulum to Golgi vesicle-mediated transport | BP | 0.00000626 | 0.00254 | 63 | 146 |
| GO:0045116 | protein neddylation | BP | 0.00000717 | 0.00254 | 14 | 18 |
| GO:0045324 | late endosome to vacuole transport | BP | 0.00006350 | 0.01500 | 11 | 14 |
| GO:0006886 | intracellular protein transport | BP | 0.00014200 | 0.02520 | 104 | 289 |
| GO:0043162 | ubiquitin-dependent protein catabolic process via the multivesicular body sorting pathway | BP | 0.00018800 | 0.02660 | 9 | 11 |
| GO:0000398 | mRNA splicing, via spliceosome | BP | 0.00029400 | 0.03360 | 59 | 150 |
| GO:0016567 | protein ubiquitination | BP | 0.00033200 | 0.03360 | 75 | 201 |
| GO:0032509 | endosome transport via multivesicular body sorting pathway | BP | 0.00056900 | 0.05040 | 9 | 12 |
| GO:0051014 | actin filament severing | BP | 0.00067200 | 0.05290 | 15 | 26 |
| GO:0043161 | proteasome-mediated ubiquitin-dependent protein catabolic process | BP | 0.00140000 | 0.09960 | 57 | 152 |

**Table S11: Annotated Genes within SNP cluster on Linkage Group 26**

| seqid | start | end | gene_id | product |
| --- | --- | --- | --- | --- |
| NC_056454.1 | 5,923,468 | 6,060,257 | LOC112224984 | striated muscle preferentially expressed protein kinase-like |
| NC_056454.1 | 6,148,929 | 6,155,404 | LOC112225090 | C-X-C chemokine receptor type 1 |
| NC_056454.1 | 6,155,544 | 6,181,267 | nabp1a | nucleic acid binding protein 1a |
| NC_056454.1 | 6,181,832 | 6,181,913 | trnas-cga_7 | tRNA-Ser |
| NC_056454.1 | 6,211,995 | 6,237,496 | cavin2a | caveolae associated protein 2a |
| NC_056454.1 | 6,266,125 | 6,408,506 | LOC112225094 | tomoregulin-2 |
| NC_056454.1 | 6,917,542 | 6,917,931 | LOC112224985 | uncharacterized mitochondrial protein AtMg00860-like |
| NC_056454.1 | 7,439,528 | 7,501,588 | slc39a10 | solute carrier family 39 member 10 |
| NC_056454.1 | 7,752,131 | 7,784,266 | esyt3 | extended synaptotagmin-like protein 3 |
| NC_056454.1 | 7,812,712 | 7,864,113 | LOC112225097 | E3 ubiquitin-protein ligase HECW2-like |
| NC_056454.1 | 7,877,001 | 7,895,144 | stk17b | serine/threonine kinase 17b (apoptosis-inducing) |
| NC_056454.1 | 7,895,117 | 7,908,200 | ftcdnl1 | formiminotransferase cyclodeaminase N-terminal like 1 |
| NC_056454.1 | 7,898,128 | 7,902,301 | lg26h2orf69 | linkage group 26 C2orf69 homolog |
| NC_056454.1 | 7,947,185 | 8,042,941 | LOC112225102 | nck-associated protein 5 |
| NC_056454.1 | 8,068,866 | 8,116,617 | LOC112225103 | ly6/PLAUR domain-containing protein 1 |
| NC_056454.1 | 8,124,721 | 8,153,662 | LOC112225104 | G-protein coupled receptor 39 |
| NC_056454.1 | 8,155,591 | 8,192,957 | LOC112225105 | solute carrier family 35 member F5 |
| NC_056454.1 | 8,242,772 | 8,264,535 | LOC112225107 | actin-related protein 3 |


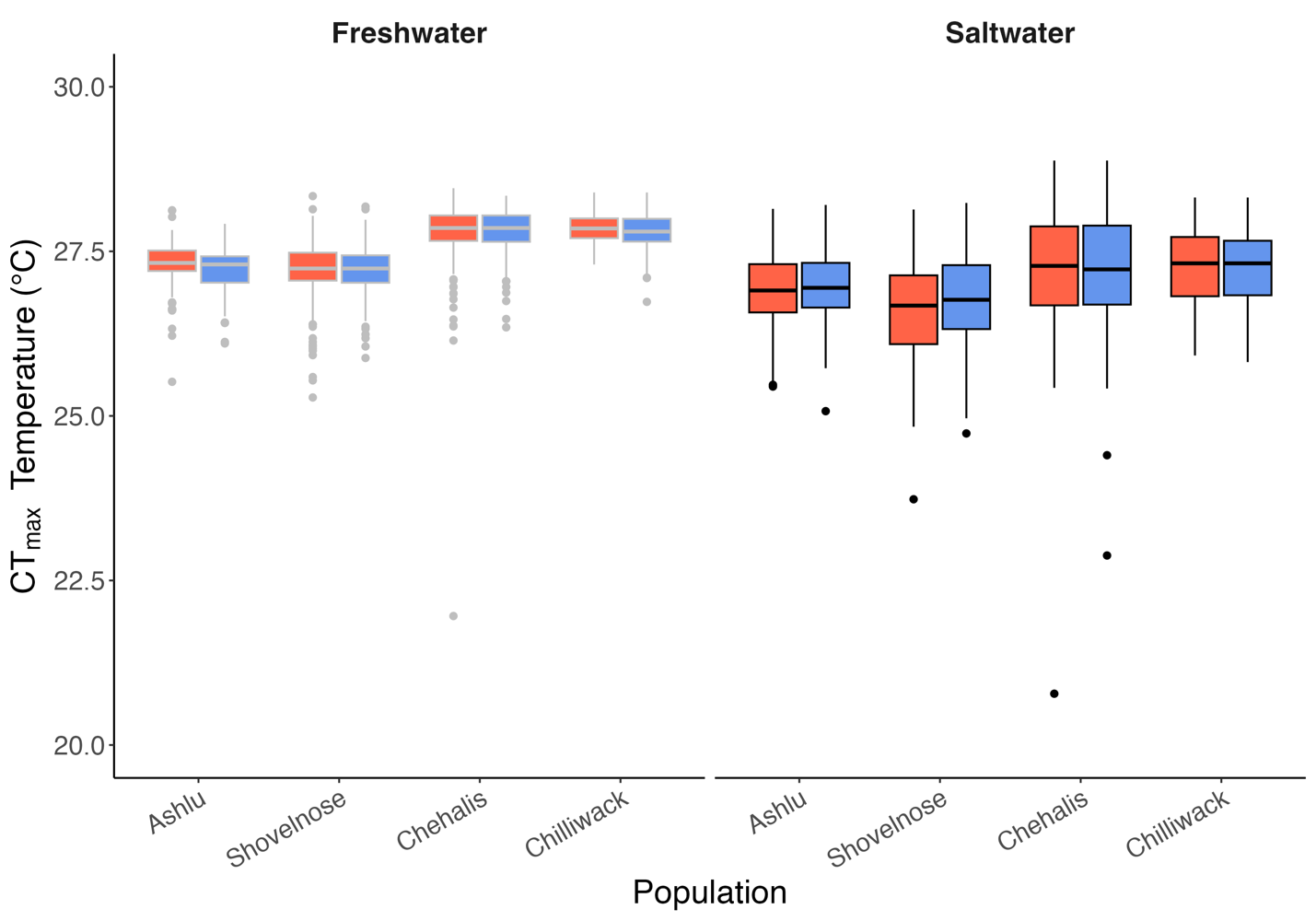


**Figure S1. Thermal tolerance (CTmax) variation across populations in freshwater and saltwater conditions, grouped by sex.**
Boxplots show the distribution of CTmax values for male (blue) and female (red) individuals across four Chinook salmon populations: Ashlu, Shovelnose, Chehalis, and Chilliwack. The y-axis represents the CTmax temperature (°C). The plot is split by salinity along the x-axis (freshwater and saltwater). Each box represents the interquartile range (IQR), with the median marked by a horizontal line. Whiskers extend to the 1.5×IQR, and outliers are shown as individual points.


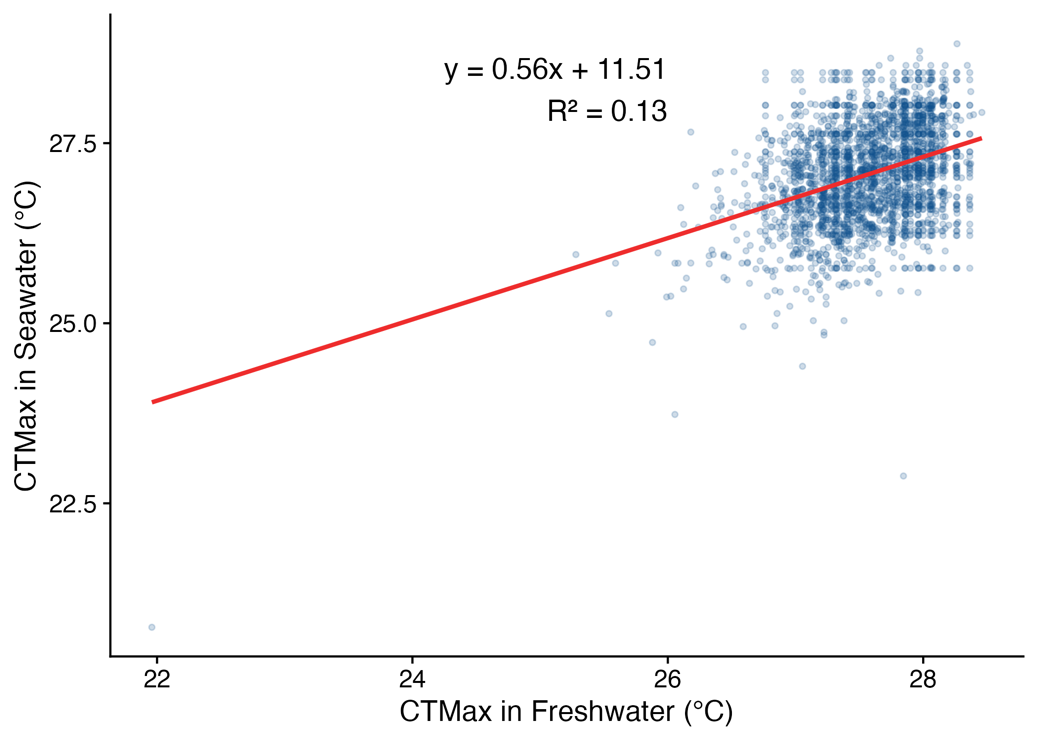


**Figure S2. Relationship between CTMax in freshwater (FW) and seawater (SW).**

Scatter plot depicting CTMax values measured in both FW and SW for the same individuals, with a linear regression line (red) illustrating the relationship. The regression equation and R² value are shown on the plot. Including the outlier increases the R² from 0.109 to 0.13; however, the correlation remains weak, indicating that thermal tolerance in FW is a poor predictor of performance in SW.


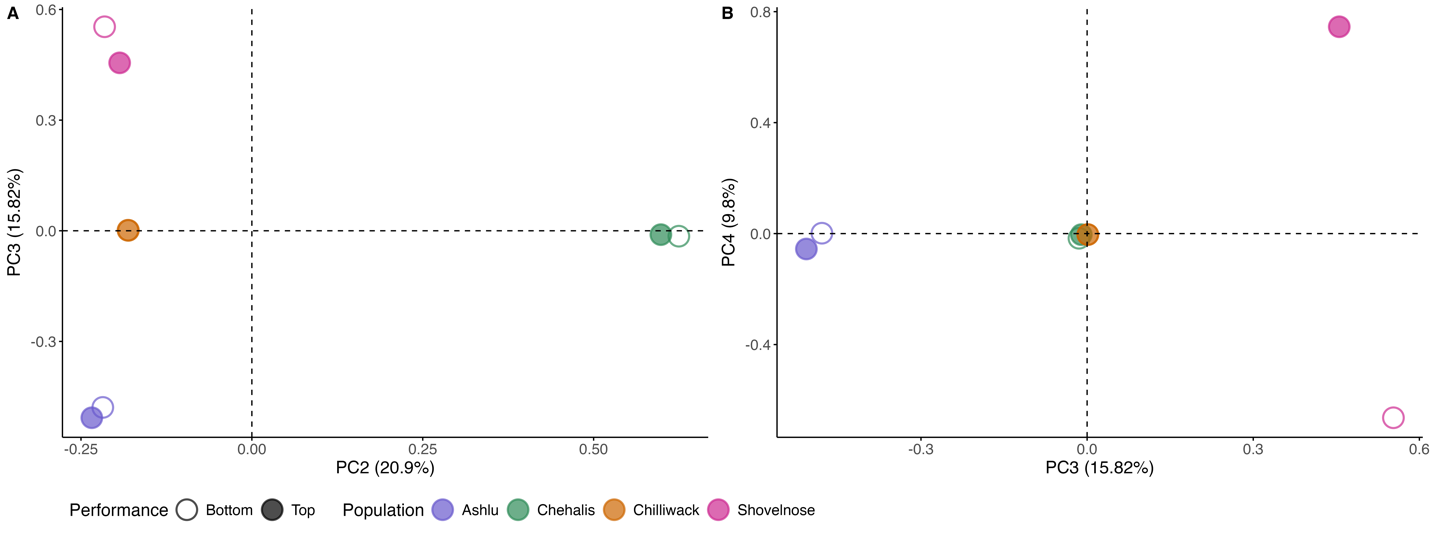


**Figure S3. Principal component analysis of Chinook salmon allele frequencies along secondary dimensions (PC2–PC4) associated with thermal tolerance.**
(A) PC2 and PC3 separate individuals by population, with no clear clustering by thermal tolerance, indicating that population-level structure remains a dominant signal in PC3. (B) PC3 captures population structure while PC4 distinguishes top- and bottom-performing individuals from the Shovelnose population, one of the two populations with a stream-type life history, representing the first axis that begins to capture variation associated with thermal performance. Each point represents a pooled sequencing sample comprising individuals from the same population and performance group, with colors indicating performance (Top vs. Bottom) and shapes denoting population of origin. Populations are distinguished by color: Ashlu (slate blue), Chehalis (green), Chilliwack (orange), and Shovelnose (magenta). Performance groups are distinguished by shape: filled symbols indicate top performers and open symbols indicate bottom performers.


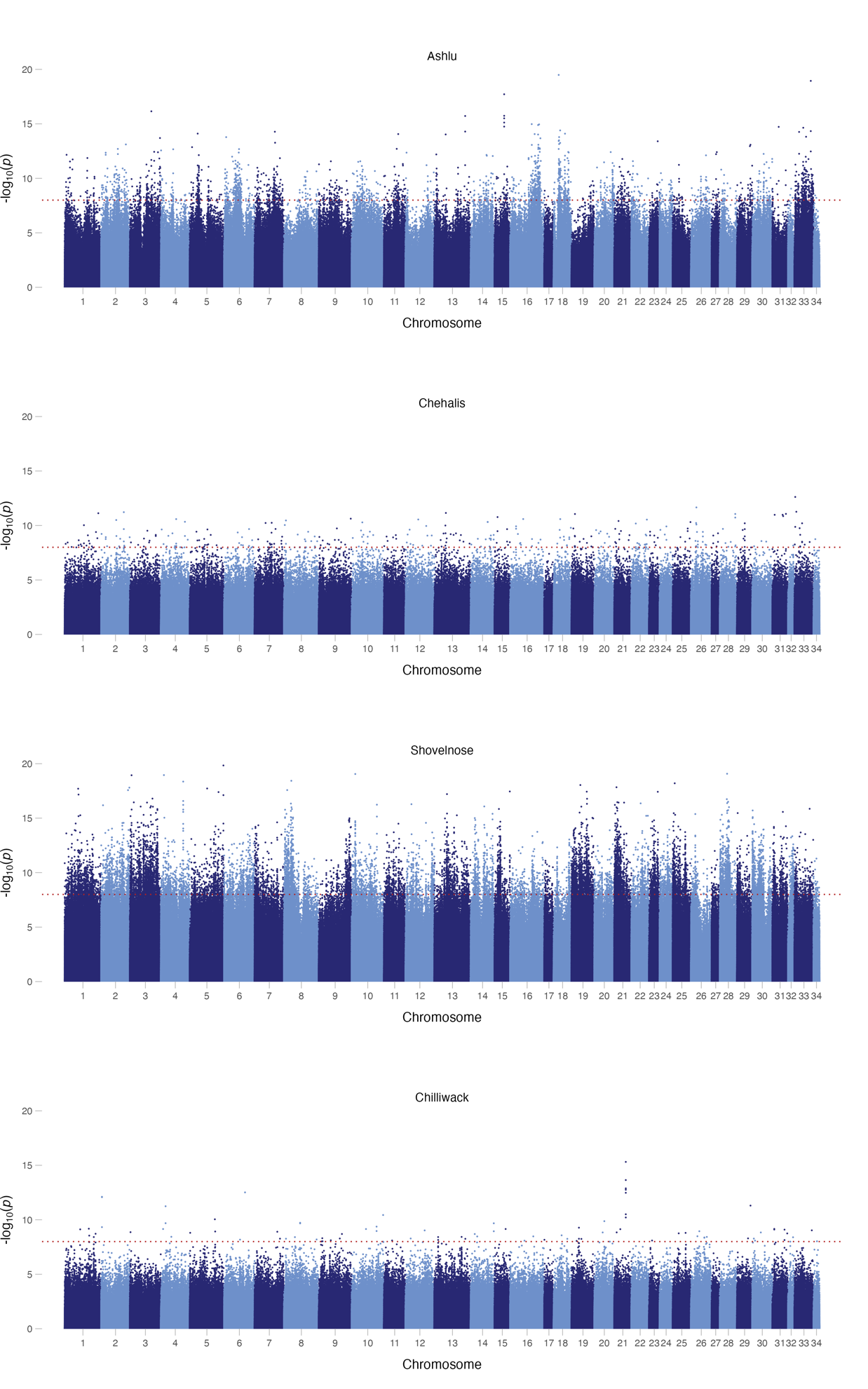


**Figure S4. Manhattan plots of –log₁₀(p) from Fisher’s exact tests comparing allele frequencies between high and low thermal performers in four Chinook salmon populations.**
Each panel shows genome-wide results from pool-seq-based differentiation analyses between the top and bottom thermal tolerance groups for a given population: (A) Ashlu, (B) Chehalis, (C) Shovelnose, and (D) Chilliwack. SNPs are plotted by genomic position across concatenated chromosomes, with alternating colors distinguishing chromosomes. The horizontal dotted line indicates the genome-wide significance threshold (–log₁₀(p) = 1e^-8^). Peaks above this threshold represent SNPs significantly differentiated between thermal performance groups, potentially reflecting loci associated with thermal tolerance.


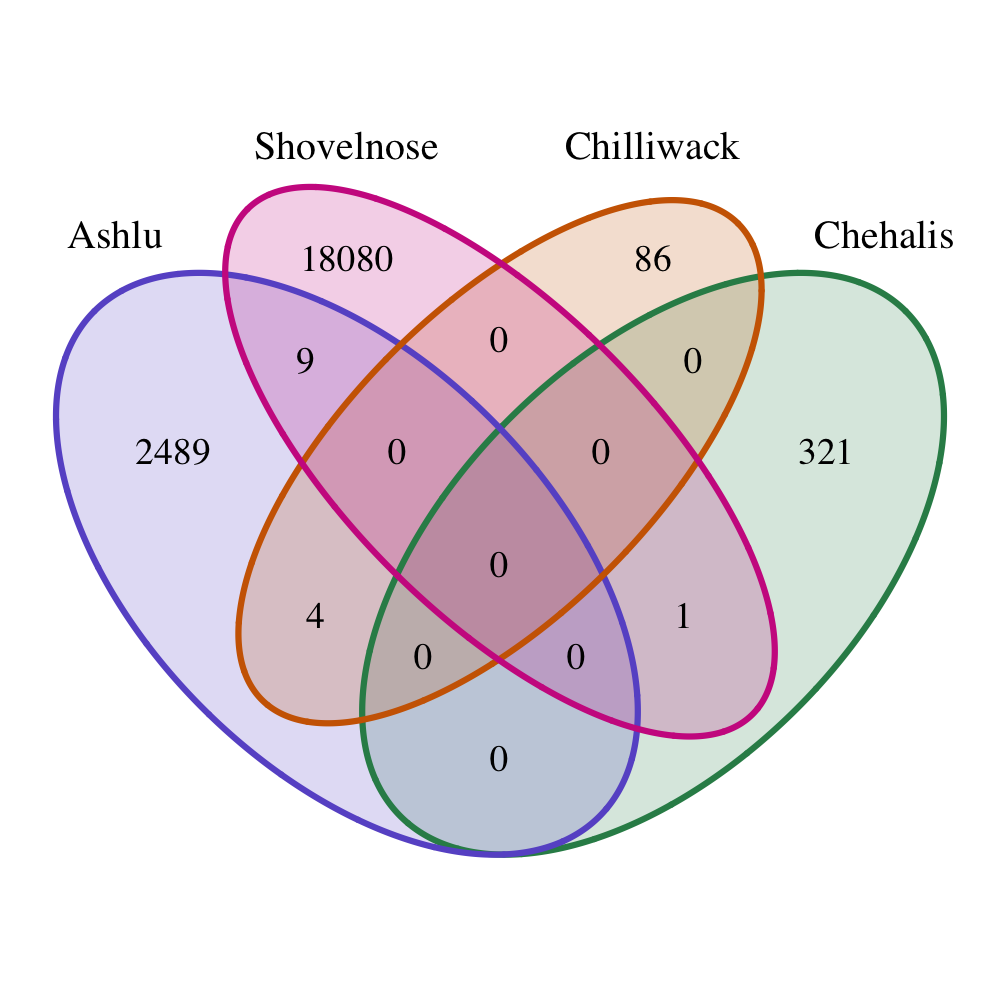


**Figure S5. Overlap of significantly differentiated SNPs between top and bottom thermal performers across four Chinook salmon populations.**
Venn diagram showing the number of unique and shared SNPs identified by Fisher’s exact tests in pool-seq data from the Ashlu, Chehalis, Shovelnose, and Chilliwack populations. SNPs were considered significantly differentiated if they exceeded the population-specific –log₁₀(p) significance threshold in the Manhattan plots (see Fig. X). Most loci are population-specific, with very limited overlap among populations, suggesting distinct genomic responses associated with thermal tolerance. Shovelnose exhibited the largest number of significant loci, while only four SNPs were shared between Chilliwack and Ashlu and a single SNP was shared between Shovelnose and Chilliwack. No loci were shared across more than two populations.


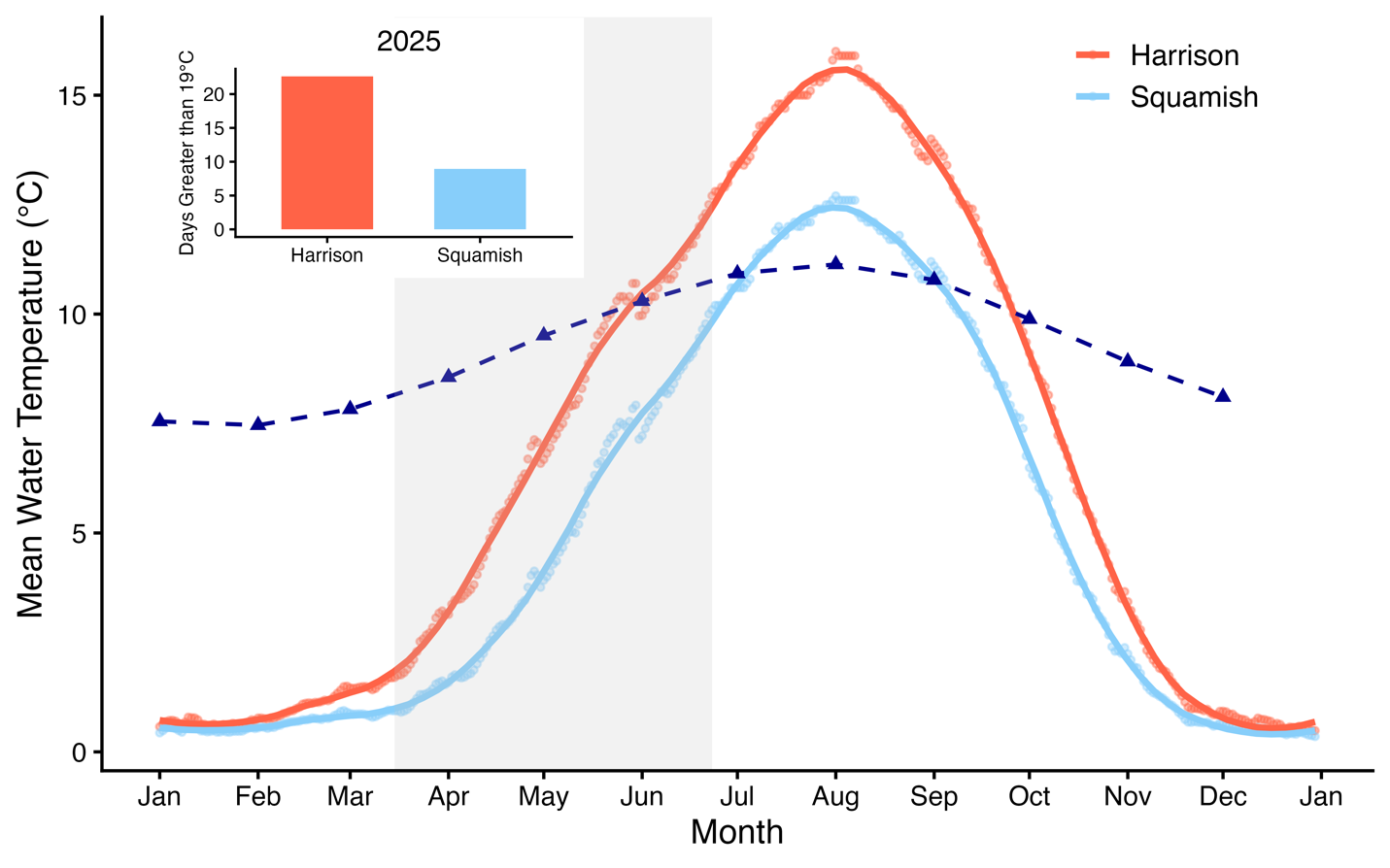


**Figure S6:** Daily mean water temperatures for the Harrison River (red) and Squamish River (light blue) from 1971–2000. Data were obtained from the Salmon Climate Impacts Portal (Pacific Climate Impacts Consortium; <https://services.pacificclimate.org/scip/app/>). Mean monthly sea surface temperatures are shown in dark blue, derived from measurements at Race Rocks Lighthouse (BC Lightstations). The shaded grey region indicates the typical freshwater outmigration period for Chinook salmon (see Wilson & Peacock, 2025, for details). Inset: number of days in 2025 that river temperatures exceeded 19 °C, based on Salmon Climate Impacts Portal data. This figure illustrates historical river thermal regimes relative to sea surface temperatures and highlights recent high-temperature events in freshwater habitats.

Wilson, S. M., & Peacock, S. J. (2025). Freshwater life-cycle timing of Pacific salmon and steelhead (*Oncorhynchus* spp.) in Canada. *Canadian Journal of Fisheries and Aquatic Sciences*, *82*, 1-17.
